# Time‐series transcriptomics and proteomics reveal alternative modes to decode p53 oscillations

**DOI:** 10.15252/msb.202110588

**Published:** 2022-03-14

**Authors:** Alba Jiménez, Dan Lu, Marian Kalocsay, Matthew J Berberich, Petra Balbi, Ashwini Jambhekar, Galit Lahav

**Affiliations:** ^1^ Department of Systems Biology Blavatnik Institute at Harvard Medical School Boston MA USA; ^2^ Laboratory of Systems Pharmacology Blavatnik Institute at Harvard Medical School Boston MA USA; ^3^ Center for Protein Degradation Dana‐Farber Cancer Institute Boston MA USA; ^4^ Ludwig Center at Harvard Medical School Boston MA USA

**Keywords:** decoding mechanisms, network motifs, p53 dynamics, proteomics, transcriptomics, Signal Transduction

## Abstract

The cell stress‐responsive transcription factor p53 influences the expression of its target genes and subsequent cellular responses based in part on its dynamics (changes in level over time). The mechanisms decoding p53 dynamics into subsequent target mRNA and protein dynamics remain unclear. We systematically quantified p53 target mRNA and protein expression over time under two p53 dynamical regimes, oscillatory and rising, using RNA‐sequencing and TMT mass spectrometry. Oscillatory dynamics allowed for a greater variety of dynamical patterns for both mRNAs and proteins. Mathematical modeling of empirical data revealed three distinct mechanisms that decode p53 dynamics. Specific combinations of these mechanisms at the transcriptional and post‐transcriptional levels enabled exclusive induction of proteins under particular dynamics. In addition, rising induction of p53 led to higher induction of proteins regardless of their functional class, including proteins promoting arrest of proliferation, the primary cellular outcome under rising p53. Our results highlight the diverse mechanisms cells employ to distinguish complex transcription factor dynamics to regulate gene expression.

## Graphical abstract

Time‐series transcriptomics and proteomics reveal that different dynamics of the transcription factor p53 generate diverse p53 target mRNA and protein expression patterns. Mathematical modeling uncovers distinct mechanisms that decode p53 dynamics.

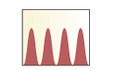

## Introduction

Cells sense changes in their internal and external environments and relay this information to the nucleus through changes in the levels or activity of proteins over time (dynamics) (Selimkhanov *et al*, 2014; Ryu *et al*, 2016; Arkun & Yasemi, 2018; Blum *et al*, 2019; Maity & Wollman, 2020). Transcription factors (TFs) are crucial for orchestrating cellular responses to stimuli. A single TF can respond to different stimuli via stimulus‐specific dynamics that help coordinate gene expression programs appropriate to the input signal. The cellular response ultimately depends on the proteins that are expressed, but in most cases, little is known about how the dynamics of the responding TF are transmitted into production of the appropriate protein effectors.

The p53 network provides a system for studying protein‐level decoding of TF dynamics. p53 is a TF that responds to a multitude of cellular stress signals including DNA damage, ribosomal dysfunction, and nutrient deprivation (Vousden & Lane, 2007; Bieging & Attardi, 2012; Boutelle & Attardi, 2021). The resulting response depends in part on the subsequent dynamical changes in p53 levels. Double‐strand DNA breaks lead to oscillatory p53 dynamics and cell cycle arrest; pharmacologically sustaining p53 expression under these conditions results in irreversible senescence or apoptosis (Purvis *et al*, 2012; Purvis & Lahav, 2013). It is not fully understood how p53 dynamics are decoded into protein‐level target gene expression, as a comprehensive analysis of the transcription and translation of p53 target genes under different dynamics has thus far not been described.

Several studies have analyzed the relationships between p53 dynamics and target gene transcription using either candidate reporters (Hanson *et al*, 2019) or global mRNA profiling (Porter *et al*, 2016; Hafner *et al*, 2017), and found that p53 target genes exhibit varied mRNA expression dynamics (Porter *et al*, 2016; Hafner *et al*, 2017; Harton *et al*, 2019). However, while transcriptional readouts are often used as a measurement of transcription factor activity, mRNA levels do not always correlate with their respective protein levels due to complex post‐transcriptional gene regulation, as well as due to dynamic changes in abundance of other regulatory proteins and cellular states (Nagaraj *et al*, 2011; Vogel & Marcotte, 2012; Koussounadis *et al*, 2015; Liu *et al*, 2016). Given that mRNA and corresponding protein levels often correlate only modestly (Schwanhäusser *et al*, 2011; Hafner *et al*, 2017; Buccitelli & Selbach, 2020), it is unclear how mRNA dynamics correspond to protein expression. One study (Hanson *et al*, 2019) analyzed select p53 targets at the protein level only under oscillatory p53 dynamics, yet a systematic view of how different p53 dynamics influence the expression profiles of its protein targets globally, as well as the underlying regulatory mechanisms, remain unknown.

In order to comprehensively investigate decoding mechanisms at both transcriptional and post‐transcriptional levels, we compared global mRNA and protein expression of p53 target genes under oscillatory or rising p53 dynamics following DNA damage. DNA damage induced by irradiation results in p53 oscillations driven by MDM2 (Lahav *et al*, 2004; Geva‐Zatorsky *et al*, 2006; Batchelor *et al*, 2011), a p53 target gene that encodes an E3 ubiquitin ligase responsible for p53 degradation (Barak & Oren, 1992; Momand *et al*, 1992; Chen *et al*, 1993). Addition of Nutlin‐3a, a small molecule inhibitor of MDM2, results in sustained p53 expression in single cells, which is manifest as rising levels when analyzed in bulk populations (Purvis *et al*, 2012). Under these conditions, we quantified global mRNA and protein levels by bulk RNA‐sequencing and tandem mass tag (TMT)‐based mass spectrometry, respectively, and analyzed the dynamic expression of p53 target genes that we have previously defined (Hafner *et al*, 2017). Analyzing the relationships between p53 dynamics and those of its target genes' transcripts and proteins revealed that oscillating p53 supported a wider diversity of target gene mRNA and protein dynamics as compared to rising p53 expression. Mathematical modeling showed that mRNA and protein degradation rates played a key role in shaping the expression profiles of a large subset of target genes. We next systemically categorized all combinations of mRNA and protein dynamics observed. We identified examples of proteins induced exclusively under rising p53 dynamics and found a variety of underlying mechanisms such as adjustment of degradation rates, adjustment of activation thresholds, or regulation by feed‐forward loops. Finally, to investigate how p53 dynamics might influence cellular outcomes, we analyzed expression of different functional classes of p53 targets. We found that expression of most classes, including those coding for proteins involved in terminal cell fates, was enhanced under rising p53 dynamics compared to oscillatory dynamics, providing a possible explanation for the increased likelihood of terminal cell fates reported under this condition (Purvis *et al*, 2012). Our results demonstrate the importance of proteomic analysis in identifying novel regulatory network motifs, as well as the significance of temporal regulation of protein expression in cellular information processing, concepts which may be extended to other proteins showing complex dynamics.

## Results

### RNA‐sequencing and TMT mass spectrometry enable quantitative analyses of p53 target genes over time

**Figure 1:**
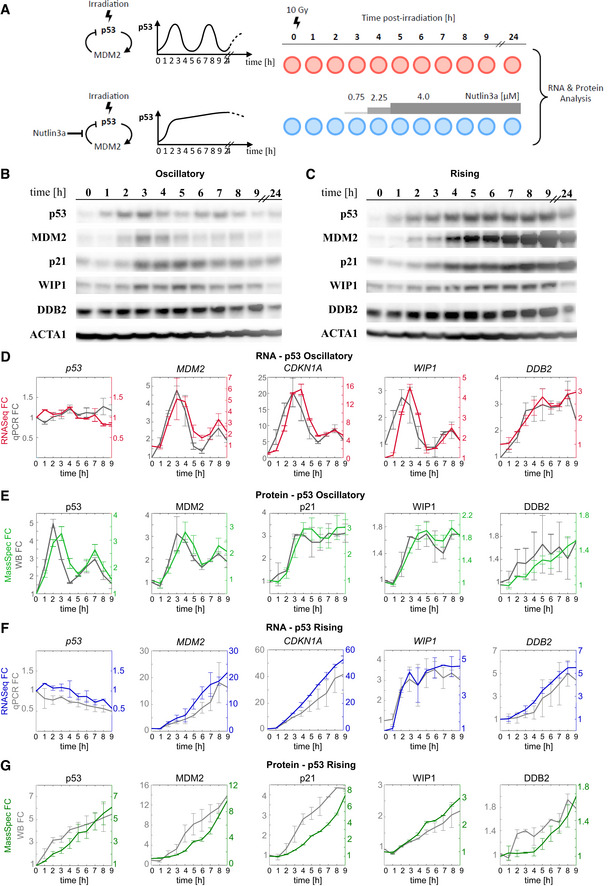
Quantification of mRNA and protein dynamics under oscillatory and rising p53 levels ASchematic showing the negative feedback loop between p53 and MDM2 and the induction of p53 oscillatory or rising dynamics using irradiation with or without the MDM2 inhibitor Nutlin‐3a, respectively. Samples for mRNA‐seq and mass spectrometry were collected at the indicated time points following radiation.B, CWestern blot for p53 and select p53 targets post‐radiation in (B) oscillatory and (C) rising conditions. Actin is shown as a loading control.D, EAverage levels of (D) mRNA quantified by RNA‐seq (red) or qRT–PCR (grey) or (E) protein quantified by mass spectrometry (light green) or western blot (grey) for select p53 target genes under oscillatory p53.F, GAverage levels of (F) mRNA quantified by RNA‐seq (blue) or qRT–PCR (grey) or (G) protein quantified by mass spectrometry (dark green) or western blot (grey) for select p53 target genes under rising p53. Data information: In (D–G), *n* = 2 biological replicates, error bars represent SD. Source data are available online for this figure.

To determine global RNA and protein expression profiles under different p53 dynamics, we established a system in which p53 levels oscillated or displayed non‐oscillatory expression. Oscillations with a period of 5.5 h were achieved by treating human cells with irradiation to induce the DNA damage response and activate the feedback loop between p53 and MDM2, the primary E3 ubiquitin ligase responsible for p53 degradation (Fig 1A) (Lahav *et al*, 2004). The oscillations persisted over the duration of the experiment (Fig 1B). p53 expression without oscillations was achieved using a previously described protocol combining radiation with incremental addition of the MDM2 inhibitor Nutlin‐3a (Purvis *et al*, 2012). This inhibitor is highly specific to MDM2 and does not induce any transcriptional changes in the absence of p53 (Tovar *et al*, 2006; Allen *et al*, 2014). Under both dynamical conditions, cells remained under cell cycle arrest and did not undergo death (Purvis *et al*, 2012; Reyes *et al*, 2018). Cells were sampled at 1‐h intervals for the first 9 h, and again at 24 h, and subjected to RNA‐seq and quantitative mass spectrometry (MS) analysis by tandem mass tag mass spectrometry (McAlister *et al*, 2014) (TMT‐MS) to obtain global transcriptional and protein expression profiles (Fig 1A). TMT‐MS allowed precise relative quantification of proteins across the two time courses of p53 dynamics. In total, 10,000 transcripts and approximately 8,000 proteins were quantified in each condition (see Materials and Methods and Data Availability section).

Global RNA and protein expression profiles determined by RNA‐seq and quantitative MS were validated for select genes using candidate‐based assays. p53 and MDM2 proteins showed the expected oscillatory dynamics in response to radiation, with the peak of MDM2 expression lagging behind the peak of p53 by approximately 1 h (Fig 1B) as described (Lahav *et al*, 2004; Geva‐Zatorsky *et al*, 2006). The second peak of p53 (at 7 h post‐radiation) was reduced in amplitude compared to its first peak (at 2 h) due to a loss of synchrony within the population of cells (Fig 1B) (Batchelor *et al*, 2008, 2011). Nutlin‐3a treatment led to continuously increasing p53 (“rising”) and MDM2 levels as well as altered dynamics of additional p53 target genes (Fig 1C). Most importantly, quantification of the mRNA and protein expression profiles of a select panel of canonical p53 targets under both dynamical conditions by real‐time quantitative PCR (Fig 1D–F) and Western blot (Fig 1E–G), respectively, showed equivalent fold changes for p53 and the examined target genes at both RNA and protein levels. Note that the mRNA of p53 itself does not change in response to radiation (Fig 1D) and the oscillations at the protein level result from regulating the stability of the p53 protein itself (Fig 1E) (Kastan *et al*, 1991; Kubbutat *et al*, 1997; Lakin & Jackson, 1999; Zhang & Chen, 2008; Shin *et al*, 2013). Overall, the high concordance between expression profiles as determined by global mRNA and protein profiles and candidate‐based assays suggests the validity of the high‐throughput measurements.

### Oscillatory p53 dynamics diversify the expression profiles of its target genes

**Figure EV1:**
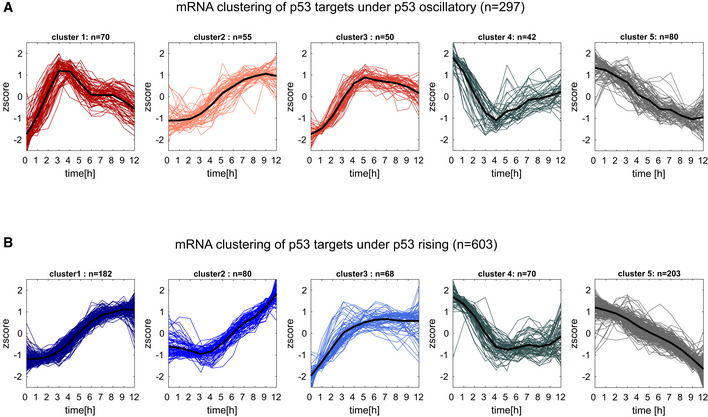
Clustering of p53 mRNA targets under oscillating and pulsatile p53 expression A, Bp53 target genes were clustered via fuzzy c‐means clustering according to their mRNA expression profiles under (A) oscillatory or (B) rising p53 expression. Differential mRNA expression was defined as fold change > 1.5 and FDR < 0.2 (*t*‐test, Benjamini–Hochberg corrected) based on two independent experiments. mRNAs were clustered based on their normalized time traces (*z* score) into five expression clusters under oscillatory and rising p53.

**Figure 2:**
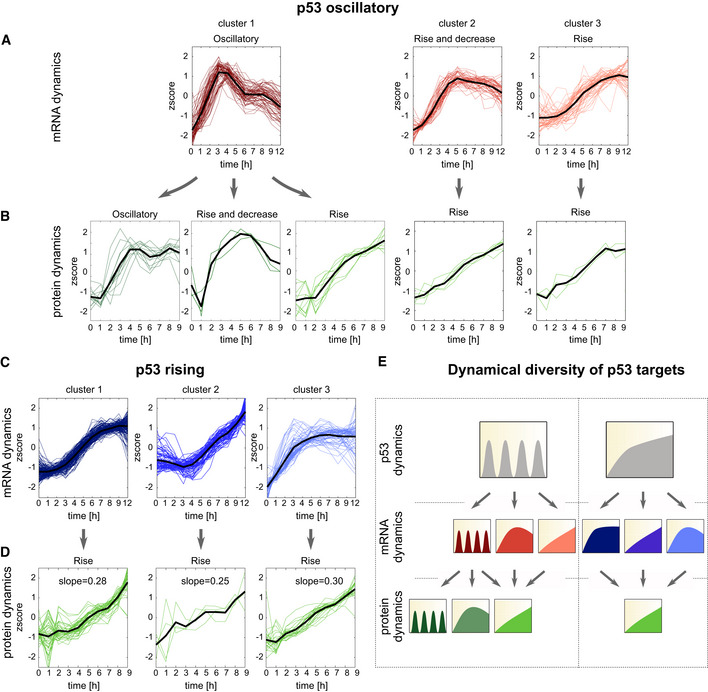
Oscillatory p53 leads to greater diversity of both mRNA and protein dynamic profiles compared to rising p53 A–DInduced p53 targets were subjected to fuzzy c‐means clustering according to their mRNA expression profiles under (A) oscillatory or (C) rising p53 expression. Differential mRNA expression was defined as fold change > 1.5 and FDR < 0.2 (*t* test, Benjamini–Hochberg corrected) based on two independent experiments. mRNAs were clustered based on their normalized time traces (*z* score) into five expression clusters under oscillatory or rising p53. Only upregulated clusters are shown. (B, D) The proteins corresponding to the mRNAs in each group were clustered according to their expression profiles. Differential protein expression was defined as fold change > 1.15 and FDR < 0.2 (*t*‐test, Benjamini–Hochberg corrected) based on two independent experiments. The mean expression of each cluster is shown in black.ESchematic showing all observed mRNA and protein dynamics under each p53 dynamical condition.

To understand how mRNA dynamics influenced protein expression, we investigated the relationships between mRNA and protein expression of p53 target genes under oscillatory and rising p53 dynamics. We focused on p53 target genes defined by the direct binding of p53 to its target promoters based on ChIP‐seq data following radiation (Hafner *et al*, 2017) (see list of target genes in Dataset EV1). From a pool of 4,141 ChIP‐seq bound p53 targets, we selected those showing robust differential mRNA expression defined as (i) false discovery rate (FDR) < 0.2 (*t*‐test, Benjamini–Hochberg corrected), (ii) fold change > 1.5 in expression relative to the basal condition at time 0 at one or more subsequent time points, and (iii) Pearson correlation between biological replicates > 0.5 (see Materials and Methods, Appendix Fig S1). Differentially expressed mRNAs yielded 297 mRNAs under oscillatory and 603 under rising conditions. We used fuzzy c‐means to cluster these transcripts into five dynamical groups based on their normalized expression (*z* score) during the first 9 h post‐irradiation under either oscillatory or rising conditions. For each condition, three clusters showed induced expression and two were repressed (Fig EV1). Because p53 is not considered to be a direct repressor of transcription (Fischer *et al*, 2014; Verfaillie *et al*, 2016; Hafner *et al*, 2017), it is likely that proteins in the repressed clusters are co‐regulated by other factors that drive repression. Thus, only induced genes, which amounted to 175 under oscillatory dynamics (Fig 2A) and 330 under rising (Fig 2C; Appendix Fig S1), were considered going forwards. These numbers align well with the number of p53 target genes identified by overlapping ChIP‐seq and expression data in other studies (Kenzelmann Broz *et al*, 2013; Moyer *et al*, 2020). Under oscillatory p53 dynamics (Fig 2A), Cluster 1 represented an oscillatory pattern, and included targets such as *MDM2*, *CDKN1A* (encoding the p21 protein), and *WIP1* that were previously shown to oscillate with p53 levels (Hafner *et al*, 2017; Hanson *et al*, 2019; Harton *et al*, 2019). The damped second oscillation of mRNAs in this cluster, as seen in Fig 1E, likely resulted from loss of synchrony of the cell population. Cluster 2 represented targets whose expression rose and then decreased, and Cluster 3 represented targets that were activated during the first 5 h and retained high expression subsequently. Under rising p53 expression (Fig 2C), Cluster 1 showed gradually rising mRNA expression, Cluster 2 showed a delayed induction followed by a continuous increase, and Cluster 3 showed rapid induction and a plateau at 4–5 h. No mRNAs showed oscillatory dynamics under Nutlin treatment, as previously noted (Hafner *et al*, 2017).

**Figure EV2:**
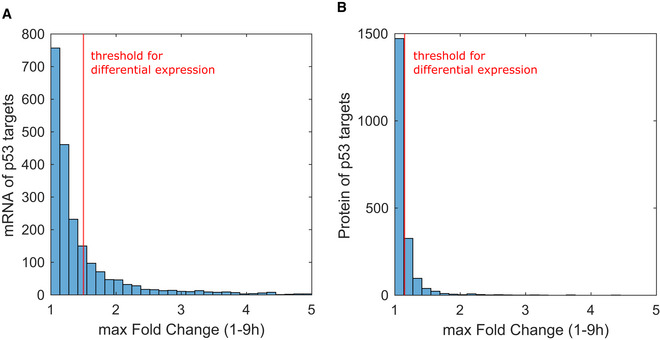
Fold change in expression of mRNA and protein levels of p53 targets A, BMaximum fold‐change (FC) distribution of mRNA and protein levels of p53 targets under both oscillatory and rising p53. Maximum fold change is calculated as the largest FC value from 1 to 9 h. Red vertical lines indicate fold‐change threshold to consider an mRNA (FC > 1.5) or protein (FC > 1.15) differentially expressed.

We next analyzed the expression of proteins corresponding to each mRNA cluster. Compared to mRNA expression levels, the corresponding fold changes in protein levels were lower, as shown by a distribution of maximum fold change in expression which was shifted to the left compared to mRNA measurements (Fig EV2). This phenomenon is not uncommon, as mRNA levels often show greater induction than their cognate proteins (Mertins *et al*, 2018; Myers *et al*, 2019). Robust differentially expressed proteins were defined as those exhibiting at least a 1.15‐fold change relative to the basal condition at one or more time points (FDR < 0.2, *t*‐test, Benjamini–Hochberg corrected, and PCC > 0.5 between biological replicates). Two house‐keeping genes that are not p53 targets, GAPDH and TUBB, were appropriately not captured by this cut‐off, and two canonical p53 targets, E2F7 and XPC, showed expression above 1.15‐fold change (Appendix Fig S2), validating our experimental approach and analysis. We noted that under both dynamical behaviors, a high percentage of induced mRNAs (65%) did not lead to induced proteins. Of the remaining mRNAs (35%) that led to induced proteins, we discovered a greater variety of protein dynamical patterns under oscillatory p53 expression compared to rising (Fig 2B–D). Protein expression under rising p53 showed uniformly induced protein dynamics independent of their mRNA dynamics, with a comparable mean slope of protein induction across all mRNA clusters (Fig 2D). Most notably, oscillatory mRNA dynamics arising from oscillatory p53 expression gave rise to a greater diversity of protein expression patterns, termed “oscillatory”, “rise”, and “rise and decrease” here (Fig 2A and B). Overall, while rising p53 expression led to a larger number of target genes being induced at both mRNA and protein levels, it limited the diversity of expression patterns. Oscillatory p53 expression induced fewer target mRNAs and proteins, but exhibited greater diversity in expression patterns of each. We therefore concluded that oscillatory p53 expression provides a greater range of possible signal relay mechanisms to expand the diversity of gene expression patterns (Fig 2E). To compare mRNA or protein trajectories under different p53 dynamics going forward, we define “detected” as those species whose expression can be measured throughout the time course under both p53 dynamical conditions. Those that rise above the threshold criteria (in relation to our untreated condition) are defined as “induced.”

### Degradation rates dominate the control of protein dynamics induced under oscillatory p53

To identify the mechanisms that regulate gene expression downstream of p53 activation, we first investigated whether a minimal model of simple regulation based on mRNA and protein synthesis and degradation rates could explain the various dynamical patterns observed above. We built a five‐parameter model that consists of two equations describing (1) mRNA dynamics and (2) protein dynamics. In order to fit mRNA expression, we used empirical measurements of p53 levels from MS data, using the values at *t*‐1 h as the input for p53 (Fig 1E). The production and degradation rate constants, kp^mRNA^ and kd^mRNA^, respectively, were fit to the RNA‐seq data. To fit protein expression, we used mRNA levels from RNA‐seq data as inputs and fit the constants for production (kp^prot^), degradation (kd^prot^), and time delay (tdel; which represents the time required for translation) to the measured mass spectrometry data of induced proteins.

(1) $$\frac{d\mathrm{mRNA}(t)}{\mathrm{dt}}={\mathrm{kp}}^{\mathrm{mRNA}}.p53\left(t‐1\right)‐{\mathrm{kd}}^{\mathrm{mRNA}}.\mathrm{mRNA}\left(t\right).$$

(2) $$\frac{d\mathrm{prot}(t)}{\mathrm{dt}}={\mathrm{kp}}^{\mathrm{prot}}\;.\;\mathrm{mRNA}\;\left(t‐\mathrm{tdel}\right)‐{\mathrm{kd}}^{\mathrm{prot}}.\mathrm{prot}\left(t\right).$$

**Figure 3:**
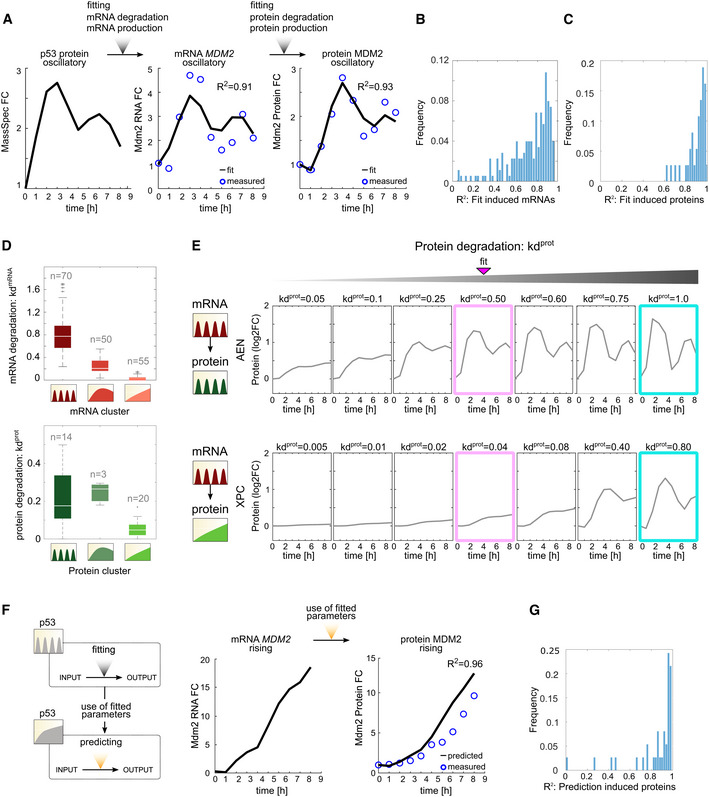
Protein degradation rates govern dynamics of p53 targets induced under oscillatory dynamics Ap53 protein levels from mass spectrometry under oscillatory dynamics were used to fit the production and degradation rates (kp^mRNA^ and kd^mRNA^, respectively) for *MDM2* mRNA. The *MDM2* mRNA levels (from RNA‐seq data in Fig 1) were used to fit the production and degradation rates (kp^prot^ and kd^prot^, respectively) for MDM2 protein. The experimentally observed and fitted measurements are shown, along with the *R* ^2^ value of the correlation between the two.B, CThe method described in (A) was used to fit parameters for all (B) mRNAs and (C) proteins induced under oscillatory dynamics. The distribution of *R* ^2^ for the fits is shown.DmRNA and protein degradation rates were calculated for each gene in Fig 2A and B, as shown in (A). The distribution of mRNA and protein degradation rates for each cluster (Fig 2A and B) under oscillatory p53 dynamics is shown. White lines indicate the median, and box edges and whiskers extend to the 25–75 and 5–95% quantiles, respectively. Individual dots represent outliers.EThe protein expression trajectories of two representative p53 target genes showing the mRNA and protein dynamics indicated at the left were modeled for the indicated values of kd^prot^. Pink box shows the value of kd^prot^ that best fits the experimentally measured protein dynamics. Cyan box shows protein expression at high kp^prot^.FThe production and degradation rates in (A) were used to predict the *MDM2* mRNA and protein expression trajectories under rising p53 dynamics. *R* ^2^ values compare the observed to expected expression patterns.GThe kp^prot^ and kd^prot^ parameters calculated for each gene in (C) under oscillatory p53 dynamics were used to predict the protein expression trajectories of the corresponding genes under rising p53 expression. The distribution of *R* ^2^ values comparing the predicted to observed expression patterns is shown.

We first fit the expression of targets that were induced both at the mRNA and protein level under oscillatory p53 dynamics. We obtained a fit with *R* ^2^ = 0.91 for mRNA expression and *R* ^2^ = 0.93 for protein expression of MDM2 (Fig 3A) and median values of *R* ^2^ = 0.83 and *R* ^2^ = 0.95 for all tested mRNAs (Fig 3B) and proteins (Fig 3C), respectively. As previously shown (Hafner *et al*, 2017), targets with oscillatory mRNA had higher kd^mRNA^ values compared to targets in non‐oscillatory mRNA clusters (Fig 3D).

**Figure EV3:**
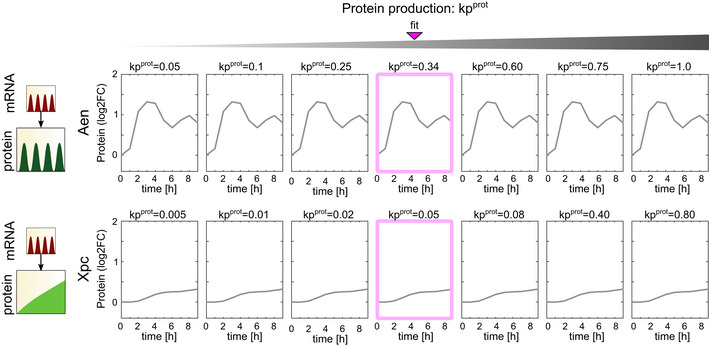
Predicted protein expression trajectories of two representative p53 target genes for different values of kp^prot^ Protein expression trajectories for targets showing the mRNA and protein dynamics indicated at the left were modeled for the indicated values of kp^prot^ (with kd^prot^ fixed at the value determined in Fig 3C). Pink box shows the value of kp^prot^ that best fits the experimentally measured protein levels.

We next tested the contribution of both the protein production and degradation rates toward shaping the protein dynamical trajectories. Because TMT‐MS data provide relative quantifications, we could not directly assess the effects of kp^prot^ or kd^prot^ on the absolute levels of protein, but we were able to analyze their effects on expression patterns. kp^prot^ had minimal effects on the pattern of relative protein expression across a range of values (Fig EV3). Similar results were reported for mRNA production rates, which did not affect dynamical patterns but did influence the magnitude of gene expression (Hafner *et al*, 2017). In contrast, kd^prot^ made a major contribution to protein expression dynamics. Oscillatory proteins showed higher degradation rates compared to rising proteins (Fig 3D), as seen for mRNAs. In general, modeling with higher kd^prot^ allowed protein expression to follow its mRNA input. By solely varying the kd^prot^ values while maintaining kp^prot^ and tdel constant in our model, we found that protein expression closely mimicked its mRNA input at high kp^prot^ (Fig 3E, cyan boxes). For example, our model predicted that as kd^prot^ rises, the naturally oscillatory protein AEN (Fig 3E top, pink box) will transition to a sharper oscillatory pattern that closely mimics the dynamics of its mRNA input (Fig 3E top, cyan box, and category a Appendix Fig S3). The same is true for the naturally slow rising XPC, which transitions to oscillatory dynamics that mimic the dynamics of its mRNA input (Fig 3E bottom and category c Appendix Fig S3). In agreement, previous experimental studies of tuning mRNA or protein stability suggested that increasing degradation rates could induce oscillations in targets that were naturally not oscillatory, as observed for MDM2 and PUMA (Hanson *et al*, 2019). In agreement with Hanson *et al*, we conclude that distinct targets' protein expression dynamics are generated depending on the relationship between target mRNA and protein stability.

To test the predictive power of the model, we next used the protein production and degradation parameters derived from the oscillatory condition to predict protein dynamics under rising p53 using RNA‐seq data as input values (Fig 3F). The predicted protein dynamics were in strong agreement with the levels measured by mass spectrometry for MDM2 (*R* ^2^ = 0.96; Fig 3F), and for the entire group of genes tested (median *R* ^2^ = 0.87; Fig 3G). Thus, the parameters (kp^prot^, kd^prot^, and tdel) that were derived from the oscillatory condition apply to expression under rising p53 dynamics. These results suggest that genes that are induced under oscillatory conditions and are further increased under rising p53 dynamics achieve this difference in expression primarily because of the differences in p53 dynamics rather than because of changes in the production or degradation rates of their mRNA or proteins.

### Identification of the simplest regulatory network motifs that decode p53 dynamics

**Figure 4:**
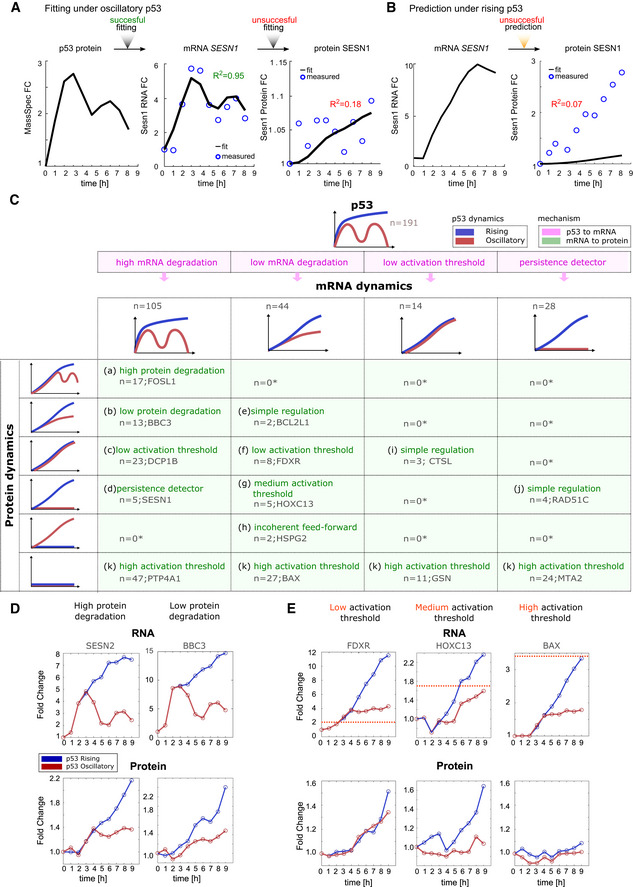
Classification of mRNA and protein dynamics suggests distinct mechanisms to decode p53 dynamics mRNA and protein production and degradation rates for SESN1 under oscillatory p53 dynamics were fit as described in Fig 3A.mRNA and protein production and degradation rates for SESN1 fitted under oscillatory dynamics were used to predict SESN1 protein expression under rising dynamics.Table showing all combinations of mRNA and protein responses of 191 genes with complete mRNA and protein measurements under both oscillatory and rising p53 dynamics. Combinations are classified into 11 different categories (a–k). An asterisk represents categories which cannot be achieved without additional complex feedback mechanisms independent of p53 dynamics. Target examples are given. Pink background represents transcriptional mechanisms while green background represents post‐transcriptional mechanisms for observed dynamics.Two targets with similar mRNA expression profiles but high (*SESN2*, category a) or low (*BBC3*, category b) protein degradation rates are shown.Examples of targets showing similar mRNA profiles but distinct protein responses resulting from distinct activation thresholds (dotted pink lines represent hypothetical threshold): low (FDXR), medium (HOXC13), and high (BAX).

**Figure EV4:**
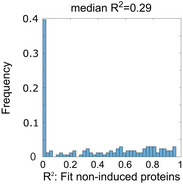
Distribution of *R* ^2^ values for the fitting of expression of p53 target proteins not induced under oscillatory conditions All proteins that were detectable but not induced under oscillatory p53 were fit for kp^prot^ and kd^prot^ based on mass spectrometry data collected under oscillatory conditions. The distribution of *R* ^2^ values for these fits is shown.

The analyses above focused on fitting production and degradation parameters to mRNAs and proteins that were induced under oscillatory p53 dynamics. We noted, however, that some proteins (e.g., SESN1) that were not induced under oscillatory dynamics became induced when p53 was rising. We first investigated whether the expression trajectories of these proteins under rising p53 expression could be predicted by fitting production and degradation parameters to their trajectories under oscillatory conditions. Under oscillatory p53, *SESN1* mRNA was induced and could accurately be fitted (*R* ^2^ = 0.95 for mRNA expression); however, SESN1 protein was not induced and therefore could not be fitted (*R* ^2^ = 0.18; Fig 4A). Therefore, it was not possible to derive k_p_ and k_d_ parameters that could be used to predict SESN1 protein levels under rising p53 expression (Fig 4B). Similar results were obtained when fitting p53 target mRNAs whose proteins were not induced under oscillatory p53 (median *R* ^2^ = 0.29; Fig EV4), suggesting that for these genes, p53 dynamics were decoded by additional mechanisms beyond intrinsic protein degradation rates.

**Figure EV5:**
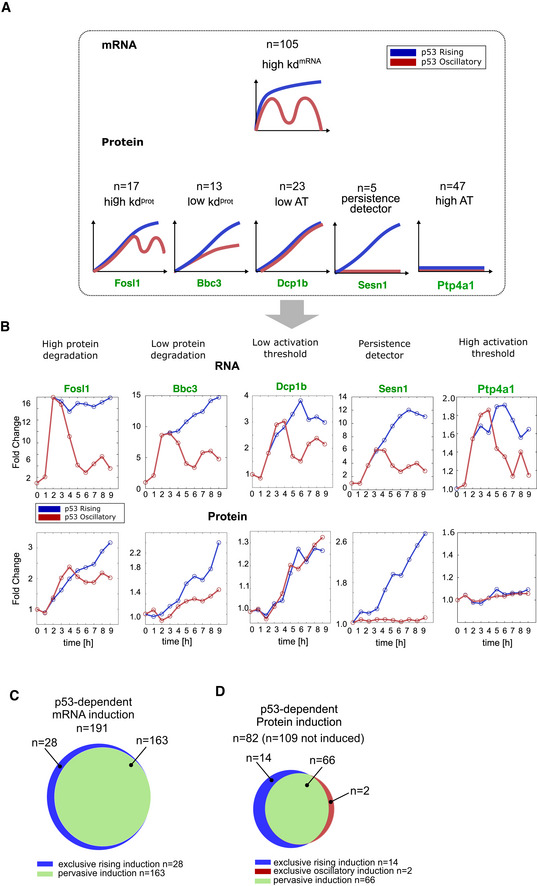
Expanded view of mechanisms decoding p53 dynamics ASchematics showing relationship between dynamical mRNA and protein responses for oscillatory mRNA.BFour targets with similar mRNA expression profiles—high mRNA degradation rate—but distinct protein profiles due to differences in protein degradation rates, activation thresholds (AT), or existence of cFFLs are shown.C, DVenn diagrams showing exclusive or pervasive induction of mRNA and protein. Fifteen percent of mRNA (28/191 = 15%) and 20% (16/82 = 20%) of proteins show exclusive induction. A total of 109 of the 191 mRNAs in (C) failed to induce proteins under either p53 dynamic.

The observation that not all p53 target genes decoded p53 dynamics based on a minimal model of mRNA and protein degradation rates prompted us to analyze whether more complex models of regulation could explain the dynamics of this class of targets (Fig 4C). To accomplish this goal, we first categorized targets according to their mRNA and protein expression patterns using empirical values derived from RNA‐seq and mass spectrometry measurements. Focusing only on mRNAs and proteins that were detected under both dynamical regimens and induced under at least one condition (Appendix Fig S1), we observed four distinct responses at the mRNA level and six at the protein level leading to 11 distinct dynamical categories (a–k; Fig 4C, full classification Appendix Fig S3). Across the four classes of mRNA dynamics, a lack of protein induction was commonly observed (Fig 4C, category k). As noted in Fig 2C, oscillating mRNA dynamics gave rise to a greater diversity of protein dynamics (Fig EV5A and B) and was the only dynamical regime that could generate oscillating protein levels. Several protein dynamical patterns—such as lack of induction or rapid induction under both oscillatory and rising p53 activation—were achieved under multiple mRNA dynamical regimes. Several combinations of mRNA and protein dynamics were not observed in our dataset. These combinations cannot be achieved without invoking additional complex feedback mechanisms independent of p53 dynamics; these are denoted by an asterisk in the table.

We used our classification to investigate the mechanisms responsible for each category of expression dynamics (a–k). Categories a, b, and e could be explained by a production–degradation minimal model in which distinct degradation rates lead to distinct dynamics. As described in Fig 3, high degradation rates for both mRNA and protein resulted in protein dynamics that mimicked p53 dynamics (Fig 4D), leading to oscillatory protein production being accessible only under oscillatory p53. The dynamics of category i could be explained by a more complex model involving an activation threshold. The concept of activation threshold (or affinity) has been previously proposed to arise through response element sequence and DNA structure (Farkas *et al*, 2021) and has been further described at the transcription level as a parameter that represents the interaction of the transcription factor with its target gene (Alon, 2007; Heltberg *et al*, 2019). A low activation threshold at the transcriptional level coupled with simple regulation via protein production/degradation rates could explain the expression of CTSL and two other proteins (category i). By analogy to the transcriptional activation threshold, a translational activation threshold could represent binding of mRNA to the translation machinery, with weakly binding mRNAs requiring higher levels to achieve protein production. The dynamics of categories c, f, g, and k could not be accurately predicted by protein production/degradation models, and instead could be explained by a post‐transcriptional activation threshold. Three targets—*FDXR*, *HOXC13*, and *BAX*—showed similar mRNA dynamics to each other under oscillatory and rising p53 dynamics (Fig 4E). However, their protein responses were quite different. FDXR protein levels were similarly induced under both oscillatory and rising p53 dynamics; HOXC13 was induced only under rising p53 input; and BAX was lowly expressed under either condition. These differences in protein levels could be explained by differences in activation threshold, with FDXR requiring minimal mRNA for protein expression, and BAX, at the other extreme, requiring high levels of mRNA. A medium activation threshold, as seen for HOXC13, provides a mechanism for exclusive expression under rising p53 dynamics (Fig 4E).

**Figure 5:**
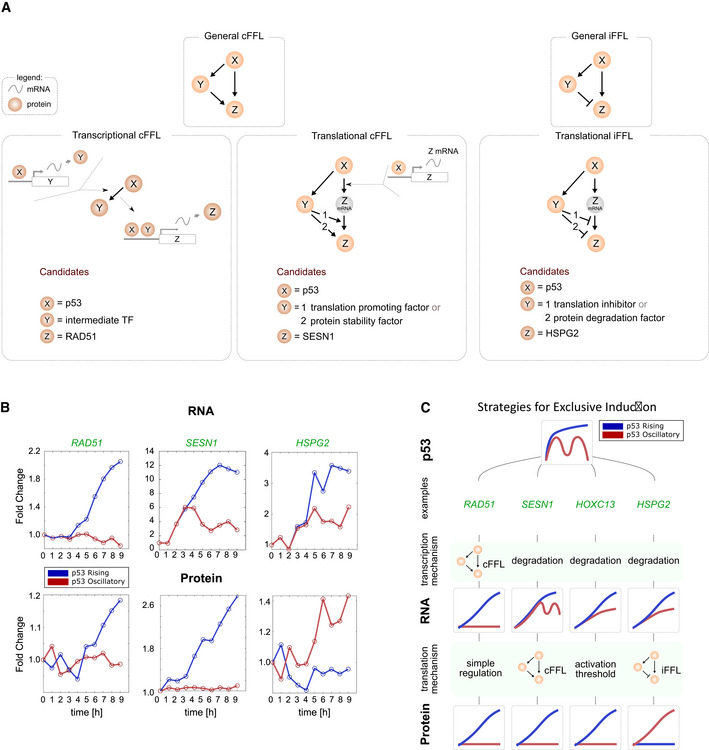
Strategies for exclusive induction under particular p53 dynamics Schematic of transcriptional and post‐transcriptional cFFLs and iFFL.Examples of genes showing exclusive protein expression under rising p53 expression due to cFFL motifs at the transcriptional (RAD51C) or translational (SESN1) levels, and exclusive protein expression under oscillatory p53 expression due to iFFL motifs at the transcriptional level (HSPG2).Four distinct strategies for exclusive protein induction.

Some categories (d, h, and j) could not be explained by models of production and degradation rates or activation threshold. We therefore investigated whether simple regulatory motifs can explain the dynamics observed. Indeed, cellular dynamic information processing described in prokaryotes reveals a range of transcriptional network motifs that perform distinct decoding functions (Mangan & Alon, 2003; Mangan *et al*, 2003; Alon, 2007). We proposed the use of feed‐forward motifs to explain the categories mentioned above. A coherent feed‐forward loop (cFFL) (Mangan & Alon, 2003; Mangan *et al*, 2003; Alon, 2007) consists of a single regulator (X) that activates both the target gene in question (Z) directly, and a second activator (Y) of the target gene (Fig 5A); both processes must occur simultaneously for the target gene to be induced. Since the activation of Y occurs with a time delay (often representing its transcription and translation), the target gene Z is activated only when the initial signal (X) persists for sufficient duration to produce Y (Fig 5A), resulting in a behavior termed “persistence detection.” In a transcriptional cFFL, Y is often a second transcription factor required for expression of the target gene Z. In a post‐transcriptional cFFL, Y could be any factor that increases the translation or the stability of the target protein. An incoherent feed‐forward loop (iFFL) consists of a single regulator (X) that directly activates the target gene (Z) and also activates a repressor (Y) of the target. When both X and Y are present, Y is epistatic and the target is not expressed (Fig 5A). Here, Y could reduce the translation or stability of the target.

Feed‐forward loops can explain the exclusive induction observed in categories d, h, and j. For example, a cFFL at the transcriptional level coupled to simple regulation of translation can result in exclusive induction of RAD51 and three other proteins under rising p53 expression (Figs 4C category j and, 5A and B). cFFL at the post‐transcriptional level coupled with high mRNA degradation rates can also result in exclusive induction under rising p53, as exemplified by SESN1 (category d, Fig 5A and B). We also found two proteins exclusively induced under oscillating p53 (Fig 4C category h, Appendix Fig S3). In both cases, their mRNAs were induced more highly under rising than oscillating p53 dynamics, leading to a counter‐intuitive inverse relationship between mRNA and protein levels (see HSPG2 panel, Fig 5B). This pattern could be explained by incoherent feed‐forward loops (Mangan & Alon, 2003; Mangan *et al*, 2003; Alon, 2007) that affect either the translation or stability of the target protein (Fig 5A).

We thus propose distinct mechanisms of increasing complexity for decoding differences in p53 dynamics at the protein level: (i) adjustment of protein production and degradation rates (Figs 3 and 4D), (ii) adjustment of activation threshold in response to an mRNA input (Fig 4E), (iii) persistence detection through the coherent feed‐forward loop (cFFL) motif at the transcriptional or translation level (Fig 5A), and (iv) inversion of mRNA to protein correlation through the incoherent feed‐forward loop (iFFL) motif (Fig 5A). Combinations of these mechanisms at transcriptional and translational levels can achieve exclusive induction under a given p53 dynamic. As noted above, coupling a transcriptional cFFL to simple regulation of translation (category j), or coupling a high mRNA degradation rate to a post‐transcriptional cFFL (category d), led to exclusive induction under rising p53 dynamics. Exclusivity in behavior could also be achieved through a precise combination of degradation rates and activation threshold (Fig 5C). The different strategies to achieve exclusive induction are portrayed in Fig 5C. Exclusive induction is a common phenomenon, with 15% of mRNAs and 20% of proteins showing exclusive induction under only one of the two dynamical regimes and remaining at constant levels under the other regime (Fig EV5C and D), highlighting the extensive dynamical decoding in p53 networks.

### Rising p53 globally enhances gene expression including anti‐proliferative programs

**Figure 6:**
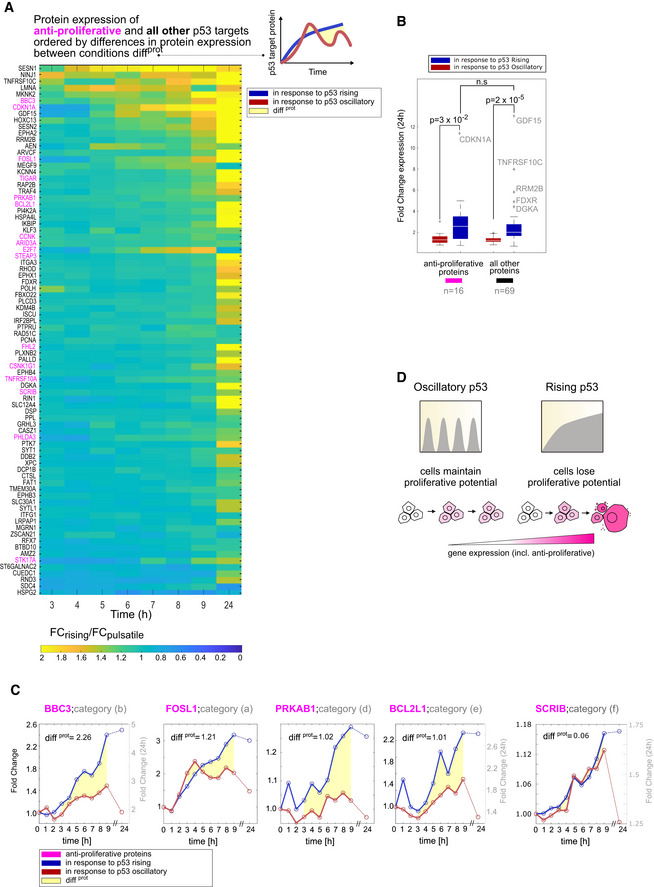
Rising p53 globally enhances the expression of genes in multiple classes Heatmap of genes ordered based on their differences in protein expression diff^prot^ between oscillatory and rising p53 (see Materials and Methods and diagram at right). Anti‐proliferative targets (those involved in apoptosis, cell cycle arrest, or senescence) are shown in pink.Boxplots showing fold changes in protein expression of anti‐proliferative proteins or all other proteins under oscillatory or rising p53 dynamics at 24 h. Central box shows median with box edges and whiskers extending to the 25–75 and 5–95% quantiles, respectively. Individual dots represent outliers. *P*‐values were calculated using a two‐sided *t*‐test. N.s., not significant.Examples of apoptotic proteins whose expression profiles fall into five distinct dynamical categories (Fig 4C, categories a, b, d, e, f). Yellow area represents differences in protein expression diff^prot^ between p53 dynamical conditions (see Materials and Methods).Enhanced expression of anti‐proliferative genes can lead to irreversible outcomes.

Finally, we investigated whether any differences in regulation of p53 targets could explain the different cellular outcomes under oscillating and rising dynamics (cell cycle arrest vs senescence and apoptosis, respectively). Focusing on proteins that were detected under both dynamical conditions, we investigated whether genes that promote cell cycle arrest, senescence, or cell death (categorized based on Gene Ontology and literature reviews (see Materials and Methods) and collectively termed “anti‐proliferative genes”) showed any common trends. In the heatmap of Fig 6A, we ordered p53 targets according to their differences in protein expression between oscillatory and rising conditions (as defined by the diff^prot^ measurement, see Materials and Methods and Fig 6A, right). We found that anti‐proliferative genes (colored pink) were spread across the heatmap (Fig 6A) and their inductions on the whole were not different from those of other functional categories of p53 target genes (Fig 6B). We also investigated whether targets involved in terminal outcomes showed common regulatory motifs, and we found that they fell into multiple classes of regulatory motifs. These results suggest that the cellular outcomes orchestrated by p53 do not rely on using similar network motifs to regulate functionally related genes, and that other mechanisms likely ensure that p53 activity does not induce conflicting cellular pathways (e.g., apoptosis and DNA repair pathways; Fig 6C). We propose that the differences in cellular outcomes between oscillatory and rising p53 dynamics arise from global enhancement of gene expression under rising conditions, allowing anti‐proliferative genes to achieve higher levels that promote terminal outcomes (Fig 6D).

## Discussion

Biological systems can encode information in the dynamical patterns of protein and mRNA expression. However, it is unclear how cells decode the information that is encoded by transcription factor dynamics into expression of appropriate target genes. In this study, we compared time‐series analyses of global p53 target mRNA and protein expression, using RNA‐sequencing and quantitative, multiplexed TMT mass spectrometry, respectively, under two different p53 dynamic regimes. These high‐throughput measurements represent the first dynamic view of the proteome downstream of p53 and provided a unique opportunity to identify decoding mechanisms both at the transcriptional and translational level. Most studies that investigate mRNA to protein correlation did so at a single time point and thus do not hold a dynamic component (Schwanhäusser *et al*, 2011; Liu *et al*, 2016; Fortelny *et al*, 2017). A few studies that monitored both mRNA and protein globally over time (Zheng *et al*, 2005; Peshkin *et al*, 2015) did so under only a single dynamical regime and did not investigate the network motifs governing mRNA and protein expression. Our integrative approach categorized all possible means by which target mRNA and protein output dynamics could be established by oscillatory and rising p53 inputs, uncovering a diverse repertoire of transcriptional and non‐transcriptional gene regulatory network motifs. While this study addressed the effects of p53 dynamics in a single cell line, we expect that strategies of p53 target regulation may be broadly applicable. A separate study also reported a diversity of p53 target gene dynamics under oscillatory conditions in RPE cell (Hanson *et al*, 2019), and the dynamics of almost all selected p53 target genes showed good concordance with our study results. Future studies across multiple cell types will be required to determine the degree of conservation of p53 target regulation on a gene‐by‐gene basis.

A multitude of cellular stresses activate p53, and one overarching question has been whether p53 can relay information on the nature of the stress by choosing which of its many effector pathways to upregulate (Zhao *et al*, 2000, 2019; Jackson & Bartek, 2009; Carvajal & Manfredi, 2013; Maier *et al*, 2016). Our results suggest that in the context of DNA damage, p53 induces many classes of its target genes— including metabolic genes, DNA repair genes, as well as cell cycle arrest and cell death effectors— indiscriminately and without enrichment for specific pathways based on its dynamics. These results are consistent with global run‐on sequencing assays showing that p53 activated target genes from multiple functional pathways (Allen *et al*, 2014). Although specific functional categories of genes were not preferentially expressed under either p53 dynamical regime, the general enhancement of gene expression under rising dynamics could explain the differences in cellular outcomes: oscillatory p53 expression leads to reversible cell cycle arrest, whereas rising p53 dynamics lead to irreversible cell senescence or cell death (Purvis *et al*, 2012). Under oscillatory dynamics, the lower levels of anti‐proliferative gene expression may not be sufficient to drive cells to terminal outcomes, allowing cells to arrest and repair DNA damage. Under rising p53 dynamics, however, the enhanced gene expression may generate sufficient anti‐proliferative activity allowing this pathway to "win" despite others also being induced (Fig 6D). A less likely alternative is that a small number of genes that are exclusively induced under one dynamical condition control cellular outcomes against a background of activation of multiple pathways. For example, PRKAB1 and SESN1 proteins, which promote autophagy by inhibiting mTOR, are induced only under rising p53 expression (Appendix Fig S3 category d, Fig 5C) and may exert anti‐proliferative effects in this condition. Conversely, CUEDC1 (Appendix Fig S3 category h), which promotes MCF7 proliferation (Lopes *et al*, 2018), is exclusively induced under oscillating p53 expression and may facilitate proliferation following DNA damage. Further studies are required to determine the potential role of these and other exclusively induced genes on cell fate. Because canonical anti‐proliferative genes do not appear to be exclusively induced, we believe it is unlikely that cell death is driven primarily by mechanisms involving exclusive gene induction under rising p53 expression. It is more likely that p53 dynamics influence cellular outcomes both by modulating levels of proteins and by regulating whether they are induced. Given that p53 oscillations are conserved across cell types and species (Stewart‐Ornstein *et al*, 2017), such dynamic information encoding may be a conserved mechanism for guiding cellular outcomes. This mechanism may allow cells extended time for completing DNA repair by prolonged maintenance of a reversible cell cycle arrest state, while simultaneously avoiding irreparable tissue damage by cell death that would arise from accumulation of apoptosis or senescence driver proteins. Future work focused on understanding events downstream of p53 activation, such as modulation of degradation rates or of expression thresholds for genes that drive different phenotypic outcomes, could elucidate how p53 dynamics coordinate with other regulatory processes to govern cellular responses.

While we and others (Hafner *et al*, 2017; Hanson *et al*, 2019) found that the dynamics of many p53 targets that were induced under oscillatory conditions were primarily controlled by degradation rates, our global analysis of gene expression under two different p53 dynamics revealed additional decoding mechanisms that, together with degradation rates, explain the entire landscape of mRNA and protein profiles. The decoding mechanisms that we observed include changes in activation threshold and use of feed‐forward network motifs. We showed that feed‐forward motifs can generate exclusive induction under oscillating (iFFLs) or rising (cFFLs) p53 expression by acting at the transcriptional and/ or translational levels. Previous studies showed that cFFLs can discriminate between a single, transient pulse versus a prolonged signal (Schleif, 2000; Shen‐Orr *et al*, 2002; Mangan & Alon, 2003; Kalir *et al*, 2005; Alon, 2007; Litvak *et al*, 2009; Gillies *et al*, 2017; Bulcha *et al*, 2019). Our study provides the first example of cFFLs decoding oscillatory dynamics. This mechanism was previously proposed to control induction of senescence under rising p53 expression (Purvis *et al*, 2012; Purvis & Lahav, 2013); however, we did not observe a particular enrichment of those motifs in the class of anti‐proliferative genes, which indicates that this regulation does not distinguish cell fates.

Although the network motifs that we identified depict the regulatory relationships between components, they do not specify the underlying molecular species. Analyses of individual genes within our dataset suggest molecular species that might achieve the observed expression patterns. The Sestrin genes (SESN1 and SESN2) (Shin *et al*, 2012; Kim *et al*, 2015) provide an example of closely related pair that shows differential regulation: SESN1 is regulated by a post‐transcriptional cFFL, while SESN2 shows simple regulation (case 1, Fig 5A). SESN1, but not SESN2, was identified as a target of the DNA damage‐responsive kinase ATM (Matsuoka *et al*, 2007), raising the possibility that ATM may participate in a translational cFFL as a factor that promotes protein stability (“Y” in the translational cFFL, Fig 5A) leading to the distinct regulation of SESN1 at the protein level.

The two targets exclusively induced under oscillatory dynamics, HSPG2 and CUEDC1, likely represent regulation by iFFLs. A common molecular mechanism for achieving such regulation in the p53 pathway involves micro‐RNAs, a family of small non‐coding RNAs that represses translation of their mRNA targets (Fig 5A). p53 induces a number of miRNAs, which were shown to act as translation inhibitory factors “Y” (Fig 5A) in iFFL networks (Hermeking, 2012). Although miRNAs targeting *HSPG2* and *CUEDC1* have not been described, exclusive induction of a different p53 target, LRP1, under sub‐lethal (low doxorubicin) but not lethal (high doxorubicin) conditions has been attributed to an miR103 (Fig 5A, “Y”) participating in a feed‐forward loop (Leslie *et al*, 2018). It is possible that a similar mechanism may hold for HSPG2 and/or CUEDC1.

Multiple motifs of high and low complexity can accomplish a given biological function (Ma *et al*, 2009; Cotterell & Sharpe, 2010; Lim *et al*, 2013; Gerardin *et al*, 2019). Minimal motifs are defined as those with the fewest interactions, and in many systems multiple minimal motifs have been identified for a given function (Ma *et al*, 2009; Cotterell & Sharpe, 2010). For example, five minimal networks, including coherent feed‐forward loop, were identified as being able to perform persistence detection (Gerardin *et al*, 2019). Of these motifs, FFLs are commonly found in organisms from bacteria (Eichenberger *et al*, 2004) and yeast (Lee *et al*, 2002) to plants and animals (Odom *et al*, 2004; Boyer *et al*, 2005). They were found to occur in *E. coli* much more often than would be expected at random (Milo *et al*, 2002; Shen‐Orr *et al*, 2002), leading us to propose these networks for explaining p53 target gene dynamics. Therefore, while we believe that the proposed minimal motifs likely operate in the p53 pathway based on their enrichment in other transcriptional networks, combinations of minimal motifs and more complex forms (with a higher number of regulatory interactions) are also candidates to explain p53 target gene dynamics.

In conclusion, our analyses revealed general principles of cellular temporal and dynamic information processing. Through unveiling diversities of network motifs, we provide empirical explanations for why mRNA and protein levels often show poor correlations in cells, thus addressing the importance of analyzing proteomics as well as surrogate transcriptomic read‐outs to map gene expression over time.

## Materials and Methods

### Cell culture

MCF7 were grown in RPMI supplemented with 10% FBS. The identity of MCF7 was confirmed by DNA fingerprinting with small tandem repeat profiling and tested negatively for mycoplasma contamination. MCF7 were irradiated with 10 Gy using a RS‐2000 X‐Ray irradiator. Nutlin‐3A was used at concentrations and times as stated (Fig 1A) following a defined protocol (Purvis *et al*, 2012).

### RNA extraction

2.5 × 10^5^ MCF7 cells per condition were subjected to cell lysis and RNA extraction including DNase I treatment according to manufacturer's protocol (Qiagen RNeasy). RNA concentrations were determined using a Nanodrop (Thermo Scientific).

### Quantitative (q)RT–PCR

One microgram of extracted RNA was used to generate complementary DNA (cDNA) using the high‐capacity cDNA reverse transcription protocol (Applied Biosystems). q‐PCRs were then performed using 1/40 of the total of cDNA, 100 nM primer, and SYBR Green reagent following the manufacturer's protocol (Applied Biosystems). Reactions were normalized to ACTA1 as a loading control.

qRT–PCR primers used:

*ACTA1* (F: TGCAGAAAGAGATCACCGC, R: CCGATCCACACCGAGTATTTG)

*BAX* (F: CTGACGGCAACTTCAACTGG, R: GATCAGTTCCGGCACCTTGG)

*CDKN1A* (F: TGTCACTGTCTTGTACCCTTG, R: GGCGTTTGGAGTGGTAGAA)

*DDB2* (F: TCATTGTTGTGGGCCGATAC, R: TGGCTCCAGATGAGAATGT

*KDM4A* (F: CGGCCAAGTCTATGGAGCC, R: TCATTGAAGCGCATGTCTGAG)

*MDM2* (F: TGCCAAGCTTCTCTGTGAAAG, R: TCCTTTTGATCACTCCCACC)

*PPDM1D* (F: ATAAGCCAGAACTTCCCAAGG, R: TGGTCAATAACTGTGCTCCTTC).

### RNA sequencing

Libraries were prepared using a SciClone G3 NGSx workstation (Perkin Elmer) using the Kapa mRNA HyperPrep kit (Roche Sequencing). Polyadenylated mRNAs were captured using oligo‐dT‐conjugated magnetic beads (Kapa mRNA HyperPrep kit, Roche Sequencing) from 300 ng of total RNA on a Perkin Elmer SciClone G3 NGSx automated workstation. Polyadenylated mRNA samples were immediately fragmented to 200–300 bp using heat and magnesium. First‐strand synthesis was completed using random priming followed by second‐strand synthesis and A tailing. dUTP was incorporated into the second strand to allow strand‐specific sequencing of the library. Libraries were enriched and indexed using 12 cycles of amplification (Kapa mRNA HyperPrep kit, Roche Sequencing) with PCR primers, which included dual 8bp index sequences to allow for multiplexing (IDT for Illumina unique dual 8bp indexes). Excess PCR reagents were removed through magnetic bead‐based cleanup using Kapa Pure magnetic beads on a SciClone G3 NGSx workstation (Perkin Elmer). Resulting libraries were assessed using a 4200 TapeStation (Agilent Technologies) and quantified by QPCR (Roche Sequencing). Libraries were pooled and sequenced on one Illumina NovaSeq SP flow cell using paired‐end, 75 bp reads.

### Western blot

Cells were lysed using RIPA buffer (Cold Springs Harbor protocols). Protein concentrations were quantified using BCA^™^ Protein Assay kit (ThermoFisher) following the manufacturer's protocol. Fifty microgram of total proteins were loaded into each lane of a 4–12% Bis–Tris gradient gel (NuPAGE), and transferred to 0.45 µm PVDF membranes (GE Healthcare). Membranes were blocked with 5% BSA, incubated with primary antibody overnight, washed and incubated with secondary HRP conjugate antibodies, followed by a last wash. Membranes were exposed using Bio‐rad Chemidoc^™^.

Primary antibodies used (all 1:1,000 unless otherwise stated): β‐ACT (Sigma‐Aldrich #A5316, 1:10,000 dilutions), BAX (CST #5023), DDB2 (CST #5416), KDM4A (CST #5328), MDM2 (Calbiochem #op46), p21 (CST #2947), p53 (Santa Cruz Biotechnology #sc‐126, 1:5,000 dilutions), and PPDM1D (Santa Cruz Biotechnology #sc‐20712). Secondary antibodies used: anti‐mouse IgG HRP linked (Invitrogen #62‐6520, 1:10,000 dilutions) and anti‐rabbit IgG HRP linked (CST #7074, 1:10,000 dilutions).

### Protein extraction, tandem mass tag (TMT) peptide labeling, and SPS MS3 analysis

Cells were lysed by addition of SDS lysis buffer (2% SDS, 150 mM NaCl, 50 mM Tris, pH 8.7) containing protease and phosphatase inhibitors (Halt^™^ Protease and Phosphatase Inhibitor Single‐Use Cocktail, EDTA Free, ThermoFisher, Catalog Number 78441). Lysate was then homogenized using Qiashredder columns (Qiagen, ref. 79656) and disulfide reduction was performed by adding dithiothreitol to a final concentration of 5 mM and heating to 37°C for 1 h, followed by alkylation with iodoacetamide at a final concentration of 15 mM followed by quenching with 50 mM DTT. Protein concentrations were determined using BCA^™^ Protein Assay kit (ThermoFisher, Catalog Number 23235). For each sample, an aliquot corresponding to 150 µg of total protein was withdrawn. Detergent was removed by methanol/chloroform protein precipitation as described previously (Wessel & Flügge, 1984). Precipitates were solubilized in 8 M urea, 20 mM EPPS, pH 8.5, and 60 µg of solubilized total protein from each sample was then used for TMT labeling. Two percent acetonitrile (v/v) was added and digestion was performed by overnight incubation in the presence of Lys‐C protease (Wako, Catalog Number 129‐02541) at an enzyme‐to‐substrate ratio of 1:75. Following further dilution of the sample with 20 mM EPPS to a final urea concentration of 0.8 M in the presence of 2% acetonitrile (v/v), digestion was performed by incubation at 37°C for 6 h with trypsin (Promega, Catalog Number V5113) at an enzyme to substrate ratio of 1:75. Equal amounts of protein were removed from each sample and labeled using a TMT11plex Mass Tag Labelling kit (ThermoFisher, Catalog Number A34808). TMT labeling efficiency and ratio checks were measured by LC–MS (Ting *et al*, 2011) analysis of a combined 11‐plex sample after combining. Equal amounts of labeled peptide from each sample were then combined for subsequent analysis. Quenching of TMT labeling reactions was performed by adding hydroxylamine to a final concentration of 0.5% (v/v) and incubating samples for 15 min at room temperature. Formic acid (FA) was added to a final volume of 2% (v/v) to lower the pH below 3.0 and samples were combined and de‐salted using a SepPak tC18 Vac RC Cartridges (50 mg, Waters, Catalog Number WAT054960). HPLC fractionation was performed using an Agilent 1200 Series instrument with a flow rate of 600 µl/min over a period of 75 min. Peptides were collected in a 96‐well plate over a 65 min gradient of 13–44%B with Buffer A comprising 5% acetonitrile, 10 mM ammonium bicarbonate, and pH 8; and Buffer B comprising 90% acetonitrile, 10 mM ammonium bicarbonate, and pH 8. Fractions were then pooled into 24 samples, followed by sample cleanup using C18 Empore^™^ Extraction Disks (Fisher Scientific, Catalog Number 14‐386‐2). The matrix was primed with methanol and equilibrated with 70% acetonitrile. For LC–MS, peptides were injected onto a 30 cm, 100 µm (inner diameter) column, and separated using an EASY‐nLC 1200 HPLC system (ThermoFisher, Catalog Number LC120). The flow rate was 450 nl/min with a gradient of 6–28%B over 240 min with first 3% acetonitrile, 0.4% FA, and then 100% acetonitrile, 0.4% FA. The column was packed with 1.8 µm C_18_ beads with a pore size of 12 nm (Sepax Technologies Inc.) heated to 60°C using a column heater. Samples from the HPLC were injected into an Orbitrap Fusion Lumos Tribrid MS (ThermoFisher, Catalog Number FSN02‐10000) using a multinotch MS^3^ method (Ting *et al*, 2011; McAlister *et al*, 2014). MS scans were performed in the Orbitrap over a scan range 400–1,400 m/*z* with dynamic exclusion. The top 10 ions with charge states from 2 to 6 were selected for MS/MS. Turbo rate scans were performed in the Ion Trap with a collision energy of 35% and a maximum injection time of 120 ms. TMT quantification was performed using synchronous precursor selection (SPS‐MS^3^) in the Orbitrap with a scan range 100–1,000 m/*z* and an HCD collision energy of 55% as described (Paulo *et al*, 2016). Assignment of MS/MS spectra was performed using the Sequest (Eng *et al*, 1994) and a Human UniProt database. Linear discriminant analysis was used to distinguish forward and reverse hits (Elias & Gygi, 2007). Peptides were identified using an MS^2^ spectrum and a FDR < 1% and was achieved by applying a target–decoy database search strategy. For protein identification and quantification, shared peptides were collapsed into the minimally sufficient number of proteins using rules of parsimony. Peptides with a total TMT value of > 200 and an isolation specificity of > 0.7 were included for quantification.

### RNA‐Seq and mass spectrometry data analysis

RNA‐seq data analysis and RNA‐seq reads were mapped and analyzed by TopHat and Cufflinks RNA‐seq analysis pipeline38, using Tophat version 2.1.0 and Cufflinks version 2.1.1. Alignment was done against the hg19 genome, and hg19 RefSeq.gtf transcript annotations were used. Selection of differentially expressed mRNAs was done by calculating the fold change and significance relative to basal expression on the two biological replicates and selecting the genes that show a (i) fold change above 1.5 with (ii) FDR (Benjamini–Hochberg) below 0.2 at any time point and (iii) Pearson's correlation between biological replicates > 0.5. Clustering on mRNA was done on *z* scores using fuzzy c‐means clustering with an exponent for the fuzzy partition matrix of 1.3 and five clusters. Selection of differentially expressed proteins was done by calculating the fold change and significance relative to basal expression on the two biological replicates and selecting the genes that show (i) a fold change above 1.15 with FDR (Benjamini–Hochberg), (ii) below 0.05 at any time point, and (iii) Pearson's correlation between biological replicates > 0.7. An additional filter was used in order to compare early differences in protein expression, with the area between pulsed and rising expression diff_early_ below 1. Clustering on cognate differentially expressed proteins was done on *z* scores using Fuzzy c‐means clustering with an exponent for the fuzzy partition matrix of 1.3 and three clusters.

Difference in protein expression between oscillatory and rising conditions during early responses (3–9 h) is defined as:

$$\mathrm{diff}=\sum_{t=3h}^{9h}1‐\frac{{\frac{\mathrm{prot}(t)}{\mathrm{prot}(t=0h)}}^{p53\;\mathrm{puls}}}{{\frac{\mathrm{prot}(t)}{\mathrm{prot}(t=0h)}}^{p53\;\mathrm{sust}}}.$$

$${\mathrm{diff}}_{\mathrm{early}}=\sum_{t=0h}^{3h}1‐\frac{{\frac{\mathrm{prot}\left(t\right)}{\mathrm{prot}\left(t=0h\right)}}^{p53\;\mathrm{puls}}}{{\frac{\mathrm{prot}\left(t\right)}{\mathrm{prot}\left(t=0h\right)}}^{p53\;\mathrm{sust}}}.$$

### Gene ontology

Gene ontology classifications were performed using AmiGO 2 (http://amigo.geneontology.org/) based on biological functions. Genes classified as “Antiproliferative” are ones which are included in any of the following three GO categories: GO:0010942—Positive regulation of cell death, GO:0045786—Negative regulation of cell cycle, and GO:2000774—Positive regulation of senescence.

### Model

Because of the discrete time points of our data, we implemented the model in Equations 1 and 2 by calculating the mRNA and protein levels at each step for a given set of (k_p_^mRNA^, k_d_^mRNA^) and (k_p_^prot^, k_d_^prot^), respectively, parameters at each time point.

$$\mathrm{mRNA}\left(t\right)\;=\left(1‐k_{d}^{\mathrm{mRNA}}\right)\;.\;\mathrm{mRNA}\left(t‐1\right)+k_{p}^{\mathrm{mRNA}}\;.\;p53(t‐1).$$

$$\mathrm{prot}\left(t\right)=\left(1‐k_{d}^{\mathrm{prot}}\right)\;.\;\mathrm{prot}\left(t‐1\right)+k_{p}^{\mathrm{prot}}\;.\;\mathrm{mRNA}\left(t‐t_{\mathrm{del}}\right).$$

We fit the model under oscillatory p53 condition as to minimize the square of the Pearson's correlation between the predicted and the measured mRNA levels for each gene.

## Author contributions

**Alba Jiménez:** Conceptualization; Formal analysis; Visualization; Writing—original draft; Writing—review and editing. **Dan Lu:** Conceptualization; Data curation; Formal analysis; Validation; Writing—original draft; Writing—review and editing. **Marian Kalocsay:** Data curation. **Matthew J Berberich:** Data curation. **Petra Balbi:** Formal analysis. **Ashwini Jambhekar:** Conceptualization; Investigation; Methodology; Writing—original draft; Writing—review and editing. **Galit Lahav:** Conceptualization; Funding acquisition; Writing—original draft; Project administration; Writing—review and editing.

In addition to the CRediT author contributions listed above, the contributions in detail are:

DL and GL conceived experiments. DL, AJi, AJa, and GL conceived analysis. DL and MJB performed experiments; MJB and MK performed MS measurements and raw MS data analysis; AJi performed analyses with advice from DL, PB, and MK; and DL, AJi, Aja, and GL wrote the study.

## Disclosure and competing interests statement

The authors declare that they have no conflict of interest. Galit Lahav is an editorial advisory board member. This has no bearing on the editorial consideration of this article for publication.

## Supporting information



Appendix

Expanded View Figures PDF

Dataset EV1

Dataset EV2

Dataset EV3

Dataset EV4

Source Data for Figure 1

## Data Availability

Proteomics raw data and search results were deposited in the PRIDE archive (Perez‐Riverol *et al*, 2019) and can be accessed under ProteomeXchange accession numbers: PXD027030 and project webpage http://www.ebi.ac.uk/pride/archive/projects/PXD027030. A list of p53 targets defined by ChIP‐seq data (Hafner *et al*, 2017) is available in Dataset EV1. RNA‐seq data containing TPM and fold change values per time point following x‐irradiation for 297 and 603 differentially expressed mRNAs under oscillatory and rising conditions, respectively, as well as their cluster assignments—numbering follows order of clusters in Fig EV1—are available in Dataset EV2. Mass spectrometry data containing TMT value and fold change values per time point for the cognate proteins of upregulated mRNA clusters under oscillatory and rising conditions as well as their protein cluster assignments (1 = oscillatory cluster, 2 = rise and decrease, 3 = rise, and 0 = not induced) are available in Dataset EV3. Fitting parameters for the cognate proteins of upregulated mRNA clusters under oscillatory condition in Fig 3 are provided in Dataset EV4. Source code for the mathematical model and RNA‐seq data are available at https://github.com/albajimenezasins/Proteomics_MSB_2022.
