# Supplementary material for: Time‐series transcriptomics and proteomics reveal alternative modes to decode p53 oscillations
Source: Mol Syst Biol. 2022 Mar 14;18(3):e10588. doi: 10.15252/msb.202110588 (PMC8919251; doi:10.15252/msb.202110588)
Supplement: Supplementary file 1 — Appendix [file MSB-18-e10588-s004.pdf]

Appendix

Time-series transcriptomics and proteomics reveal alternative modes to decode p53 oscillations

Alba Jimenez*[,1], Dan Lu*[,1], Marian Kalocsay[,1,2], Matthew J. Berberich[,2,3], Petra Balbi[,1], Ashwini Jambhekar**[,1], Galit Lahav**[,1]

*=authors with equal contributions

**= co-corresponding authors

1. Department of Systems Biology, Blavatnik Institute at Harvard Medical School, Boston, MA 02115, USA
2. Laboratory of Systems Pharmacology, Blavatnik Institute at Harvard Medical School, Boston, MA 02115, USA
3. Center for Protein Degradation, Dana-Farber Cancer Institute, Boston, MA 02115

Correspondence: galit@hms.harvard.edu / ashwini_jambhekar@hms.harvard.edu

Table of content

Appendix Figure S1. Flowcharts detailing the different data sets and filters used in the study.

Appendix Figure S2. mRNA and protein profiles of house-keeping genes GAPDH and TUBB, and p53 targets E2F7 and XPC.

Appendix Figure S3. mRNA and protein profiles of all targets classified into the 11 categories of dynamical responses.

Appendix Figure S1

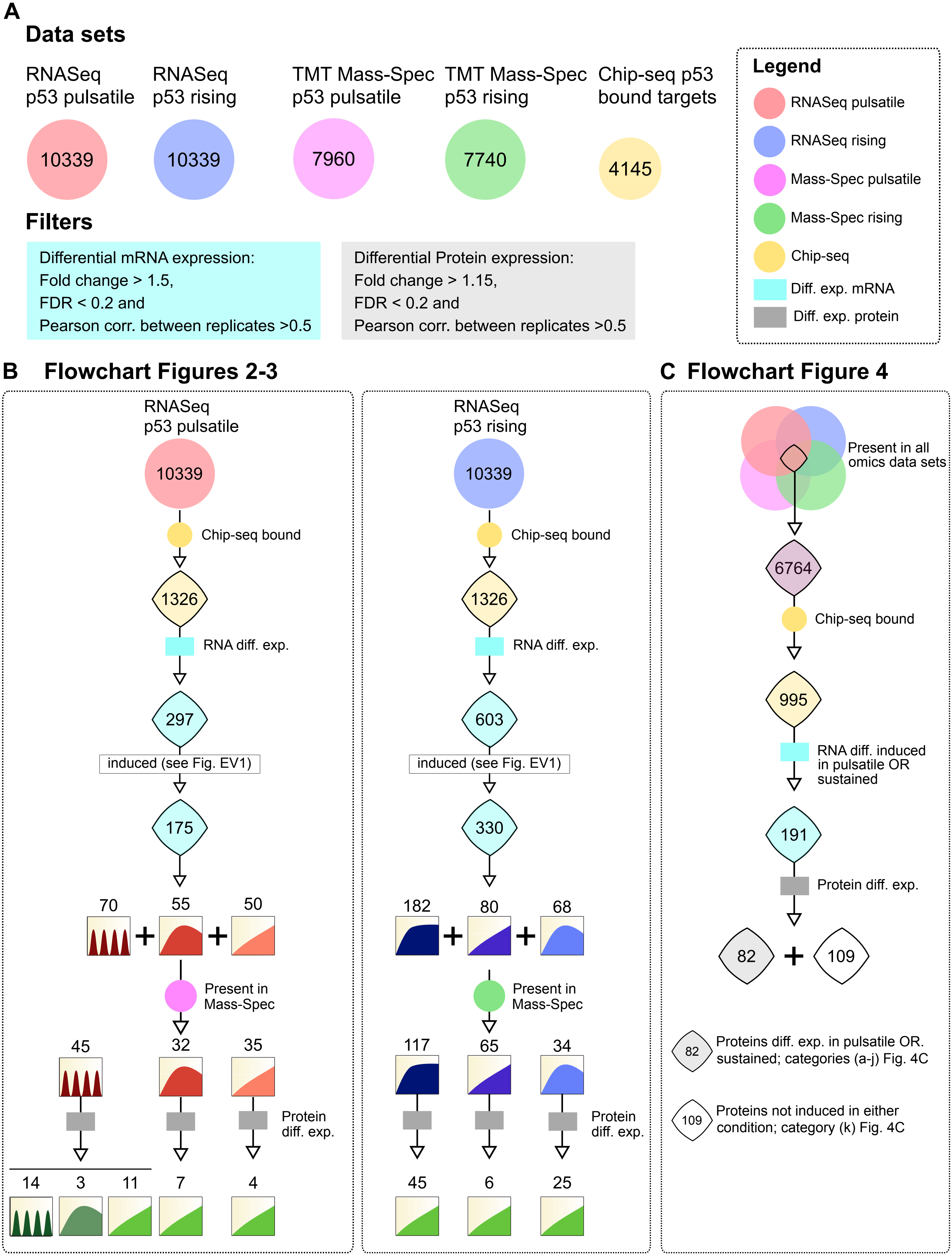

Appendix Figure S1: Flowcharts detailing (A) The different data sets and filters used in the study (B) Flowcharts for Figures 2 and 3 (C) Flowchart for Figure 4.

Appendix Figure S2

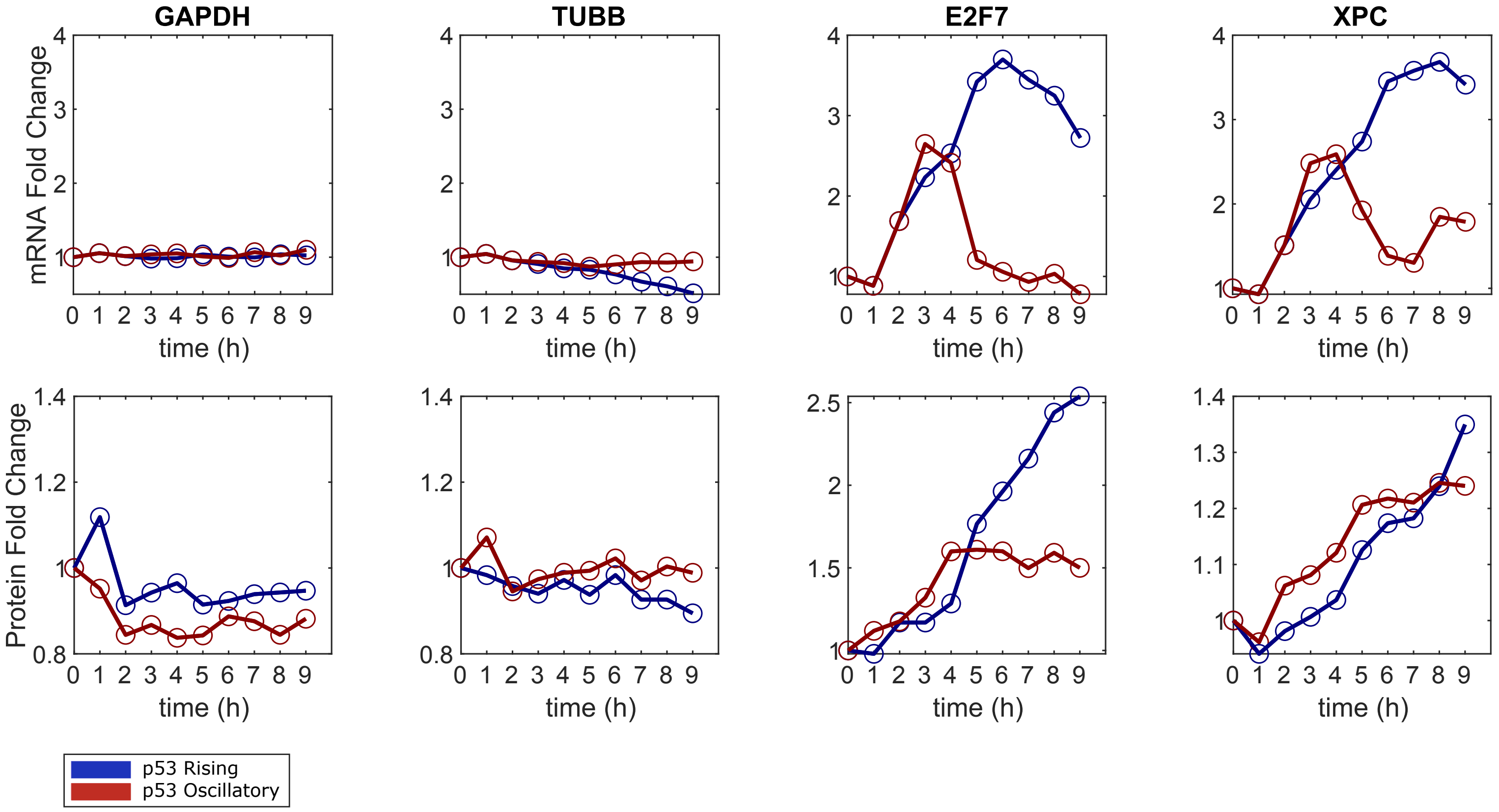

Appendix Figure S2: mRNA and protein profiles of house-keeping genes GAPDH and TUBB, as well as two p53 targets E2F7 and XPC.

Appendix Figure S3

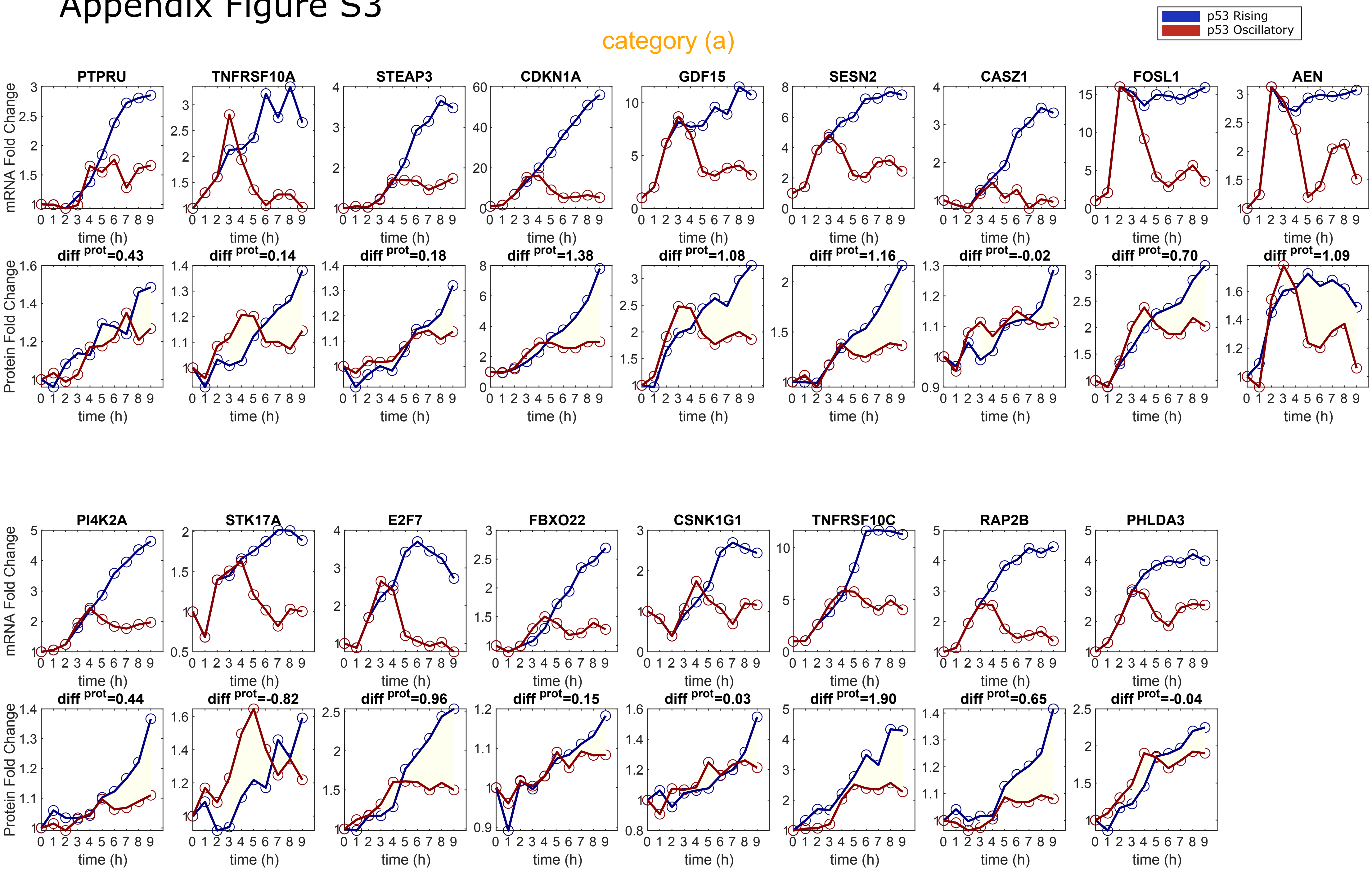

Appendix Figure S3: mRNA and protein profiles of all targets classified into 11 categories (a-k) of dynamical responses as defined in Figure 4.

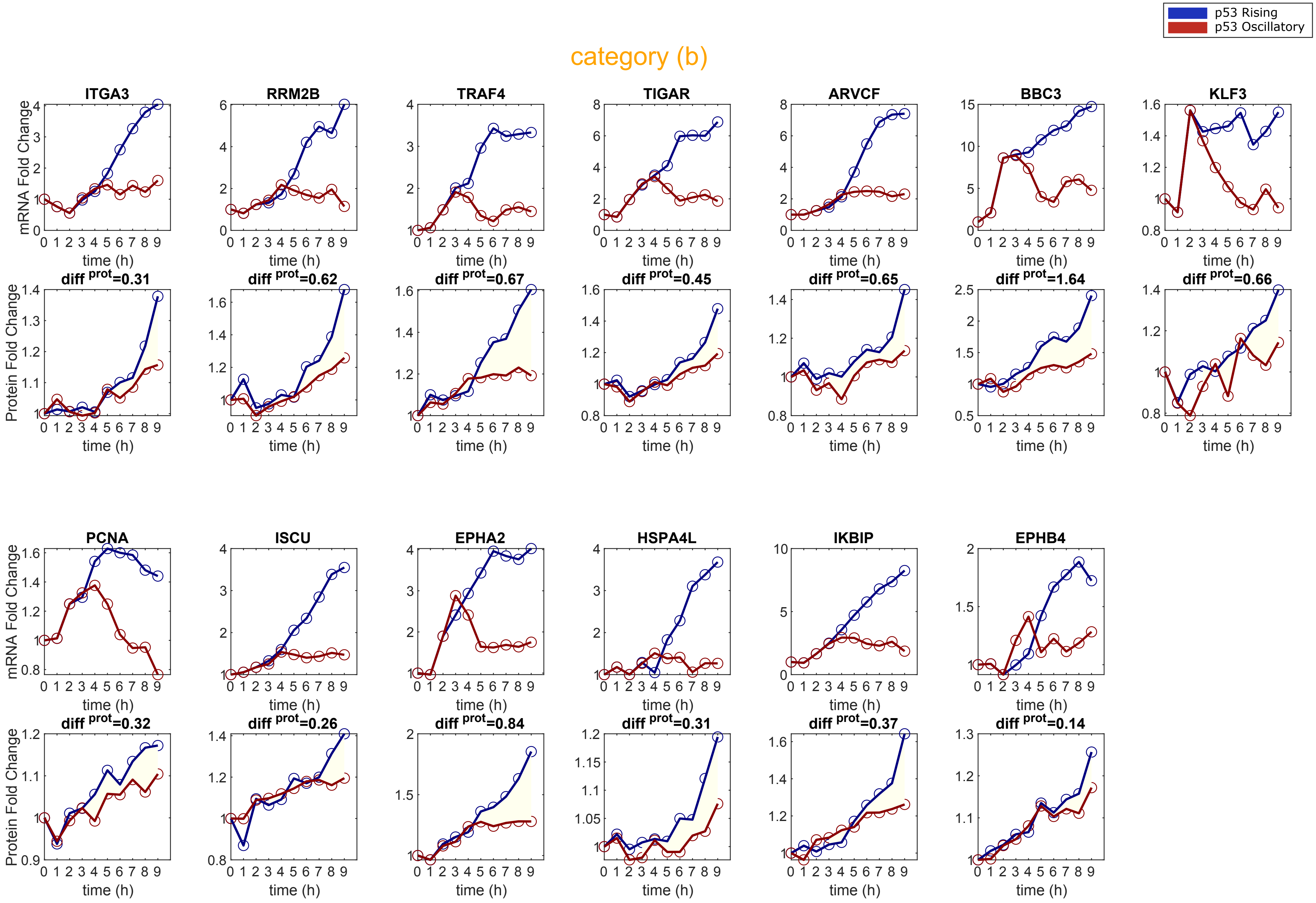
p53 Rising
p53 Oscillatory
category (b)
mRNA Fold Change
Protein Fold Change
time (h)
ITGA3
RRM2B
TRAF4
TIGAR
ARVCF
BBC3
KLF3
diff prot=0.31
diff prot=0.62
diff prot=0.67
diff prot=0.45
diff prot=0.65
diff prot=1.64
diff prot=0.66
PCNA
ISCU
EPHA2
HSPA4L
IKBIP
EPHB4
diff prot=0.32
diff prot=0.26
diff prot=0.84
diff prot=0.31
diff prot=0.37
diff prot=0.14

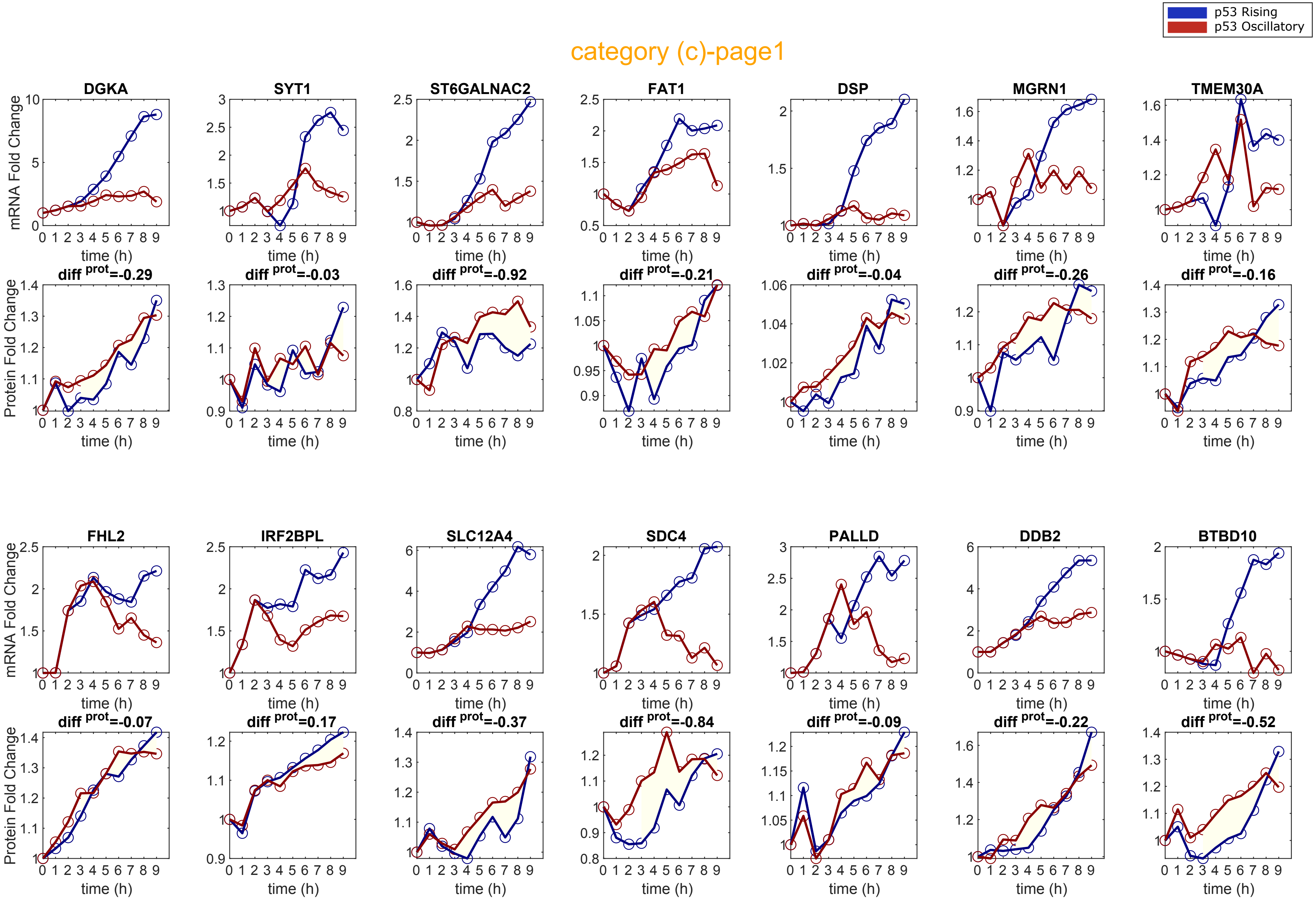
p53 Rising
p53 Oscillatory
category (c)-page1
DGKA
SYT1
ST6GALNAC2
FAT1
DSP
MGRN1
TMEM30A
mRNA Fold Change
time (h)
diff prot=-0.29
diff prot=-0.03
diff prot=-0.92
diff prot=-0.21
diff prot=-0.04
diff prot=-0.26
diff prot=-0.16
Protein Fold Change
FHL2
IRF2BPL
SLC12A4
SDC4
PALLD
DDB2
BTBD10
diff prot=-0.07
diff prot=0.17
diff prot=-0.37
diff prot=-0.84
diff prot=-0.09
diff prot=-0.22
diff prot=-0.52

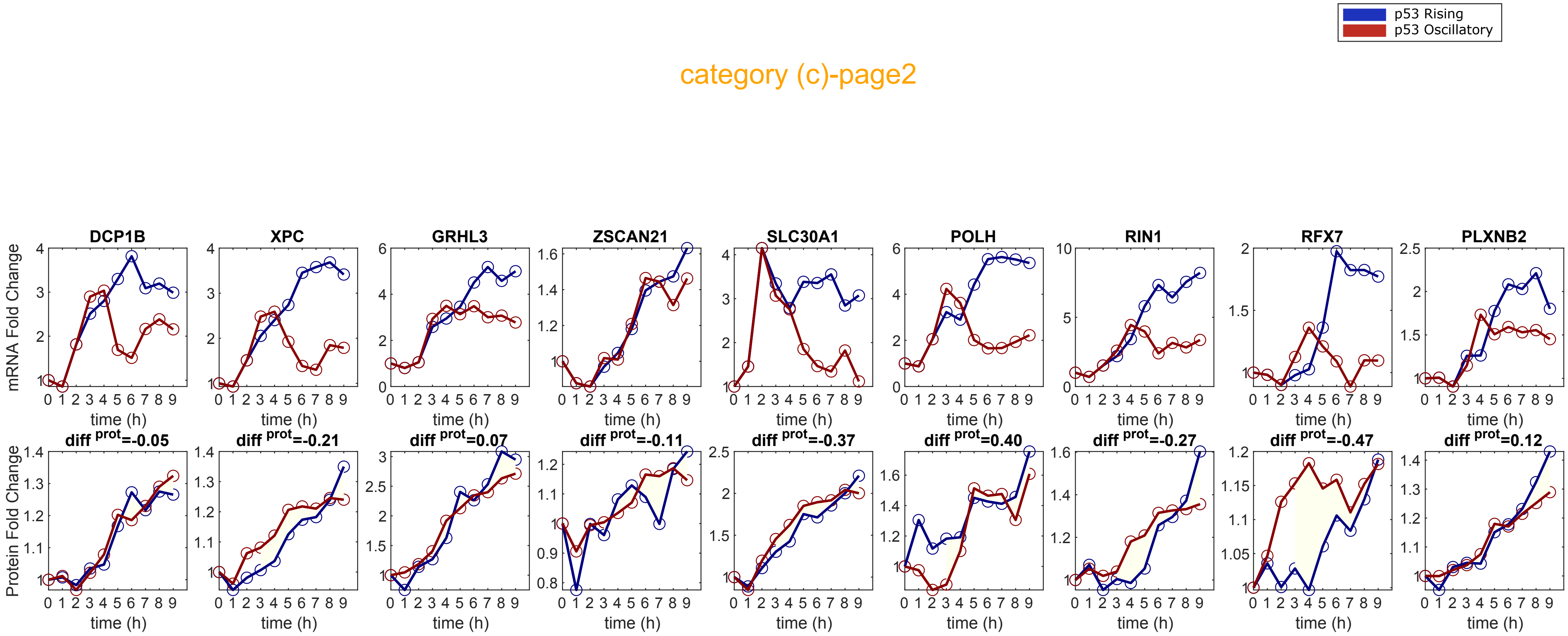

p53 Rising
p53 Oscillatory
category (c)-page2
DCP1B
XPC
GRHL3
ZSCAN21
SLC30A1
POLH
RIN1
RFX7
PLXNB2
mRNA Fold Change
Protein Fold Change
time (h)
diff prot=-0.05
diff prot=-0.21
diff prot=0.07
diff prot=-0.11
diff prot=-0.37
diff prot=0.40
diff prot=-0.27
diff prot=-0.47
diff prot=0.12

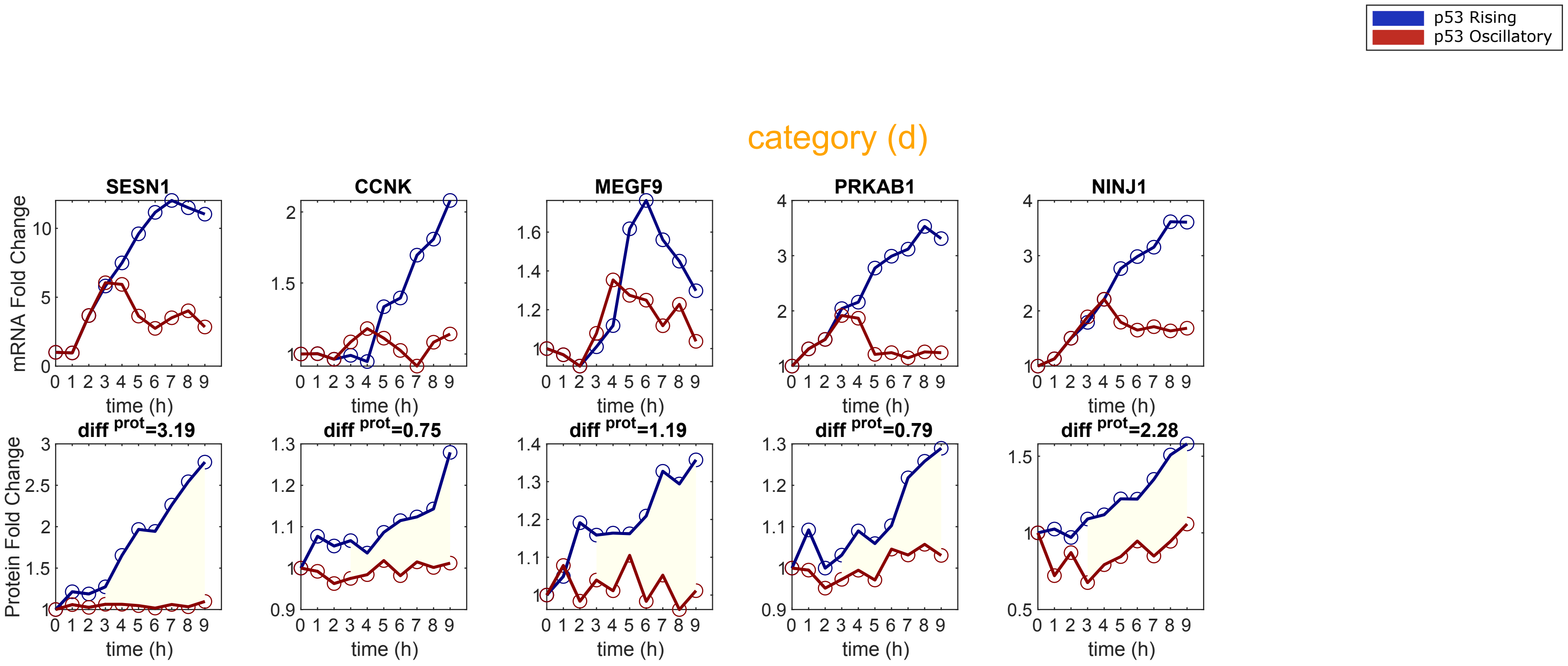
p53 Rising
p53 Oscillatory
category (d)
SESN1
CCNK
MEGF9
PRKAB1
NINJ1
mRNA Fold Change
Protein Fold Change
time (h)
diff prot=3.19
diff prot=0.75
diff prot=1.19
diff prot=0.79
diff prot=2.28

category (e)

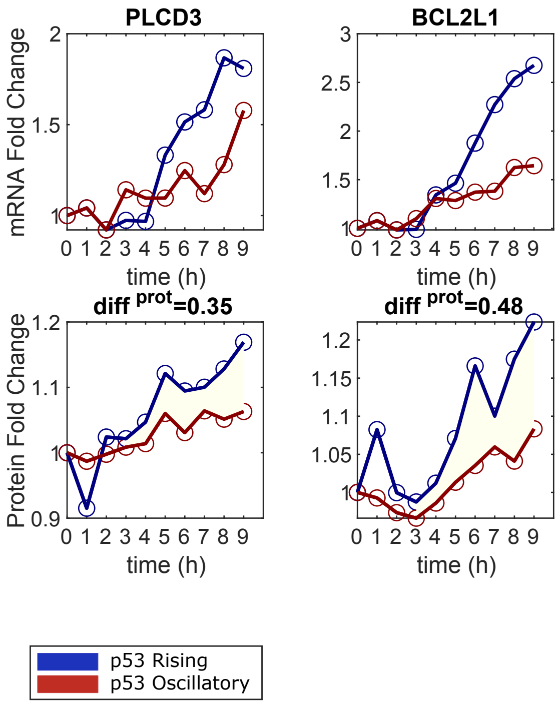
PLCD3
BCL2L1
mRNA Fold Change
Protein Fold Change
time (h)
diff prot=0.35
diff prot=0.48
p53 Rising
p53 Oscillatory

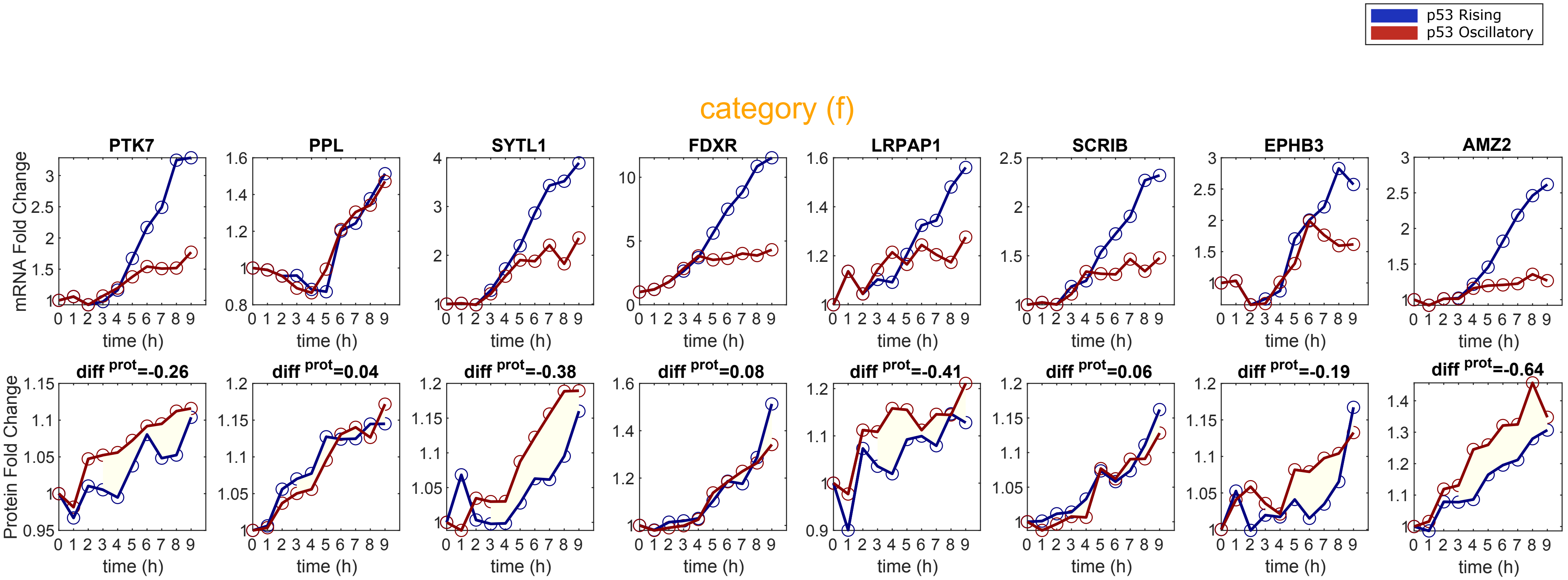
p53 Rising
p53 Oscillatory
category (f)
PTK7
PPL
SYTL1
FDXR
LRPAP1
SCRIB
EPHB3
AMZ2
mRNA Fold Change
Protein Fold Change
time (h)
diff prot=-0.26
diff prot=0.04
diff prot=-0.38
diff prot=0.08
diff prot=-0.41
diff prot=0.06
diff prot=-0.19
diff prot=-0.64

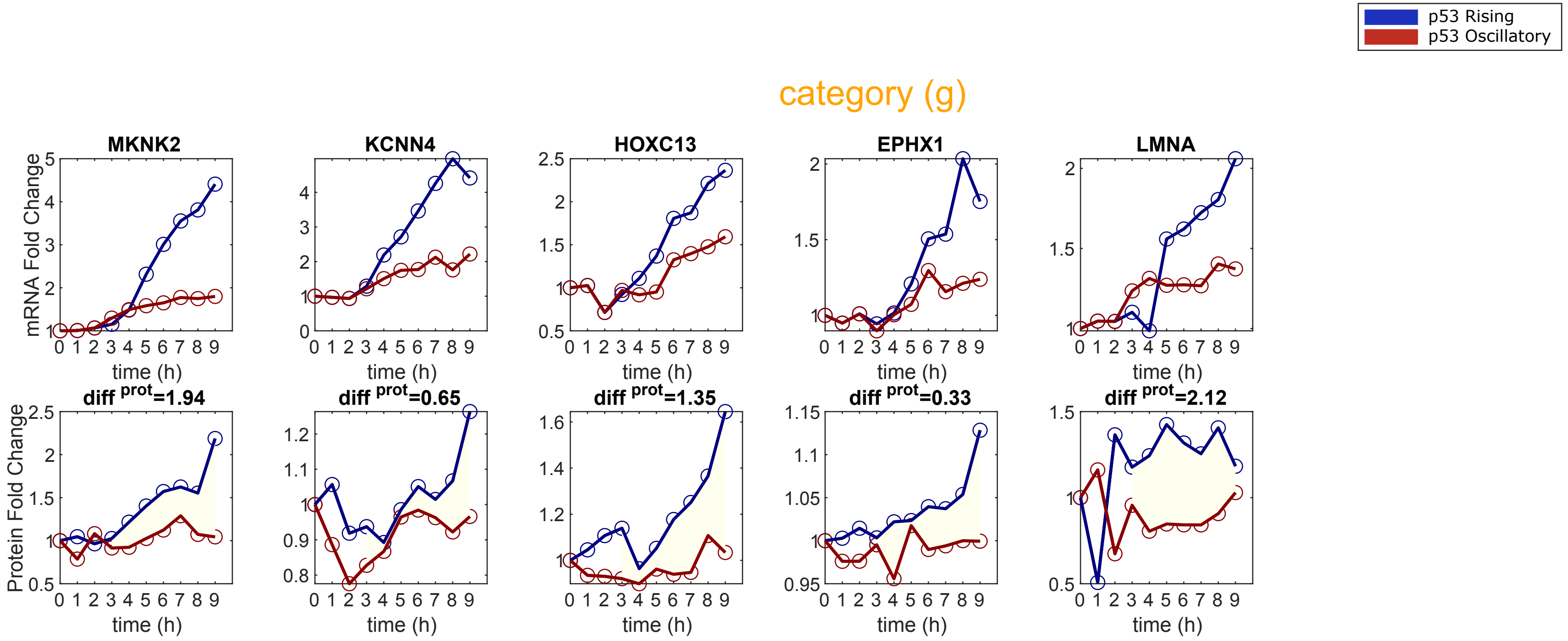
p53 Rising
p53 Oscillatory
category (g)
MKNK2
KCNN4
HOXC13
EPHX1
LMNA
mRNA Fold Change
Protein Fold Change
time (h)
diff prot=1.94
diff prot=0.65
diff prot=1.35
diff prot=0.33
diff prot=2.12

category (h)

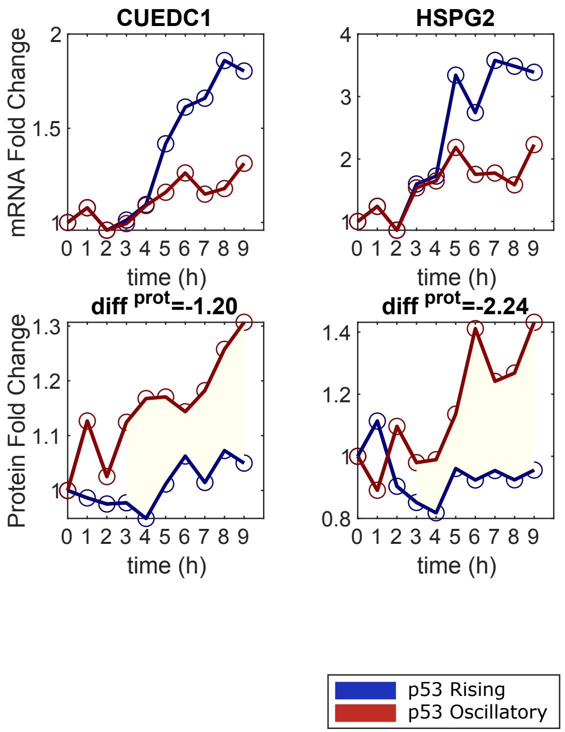

CUEDC1
HSPG2
mRNA Fold Change
time (h)
diff prot=-1.20
diff prot=-2.24
Protein Fold Change
p53 Rising
p53 Oscillatory

category (i)

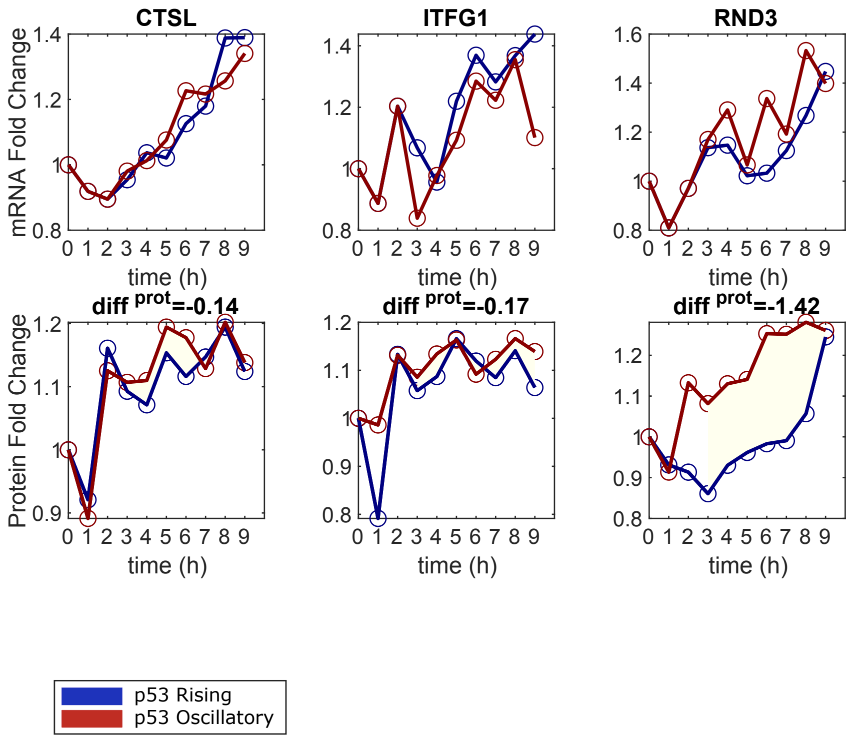

CTSL
ITFG1
RND3
mRNA Fold Change
Protein Fold Change
time (h)
diff prot=-0.14
diff prot=-0.17
diff prot=-1.42
p53 Rising
p53 Oscillatory

category (j)

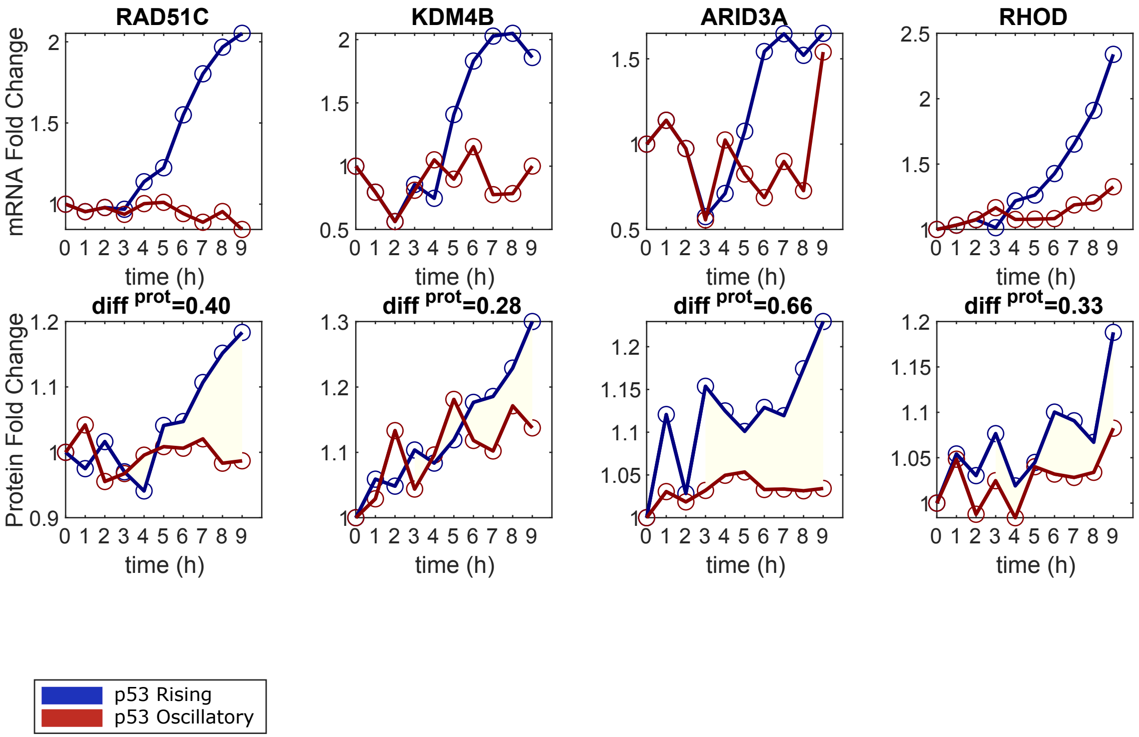

RAD51C
KDM4B
ARID3A
RHOD
mRNA Fold Change
Protein Fold Change
time (h)
diff prot=0.40
diff prot=0.28
diff prot=0.66
diff prot=0.33
p53 Rising
p53 Oscillatory
