# Supplementary material for: Time‐series transcriptomics and proteomics reveal alternative modes to decode p53 oscillations
Source: Mol Syst Biol. 2022 Mar 14;18(3):e10588. doi: 10.15252/msb.202110588 (PMC8919251; doi:10.15252/msb.202110588)
Supplement: Supplementary file 2 — Expanded View Figures PDF [file MSB-18-e10588-s005.pdf]

Expanded View Figures

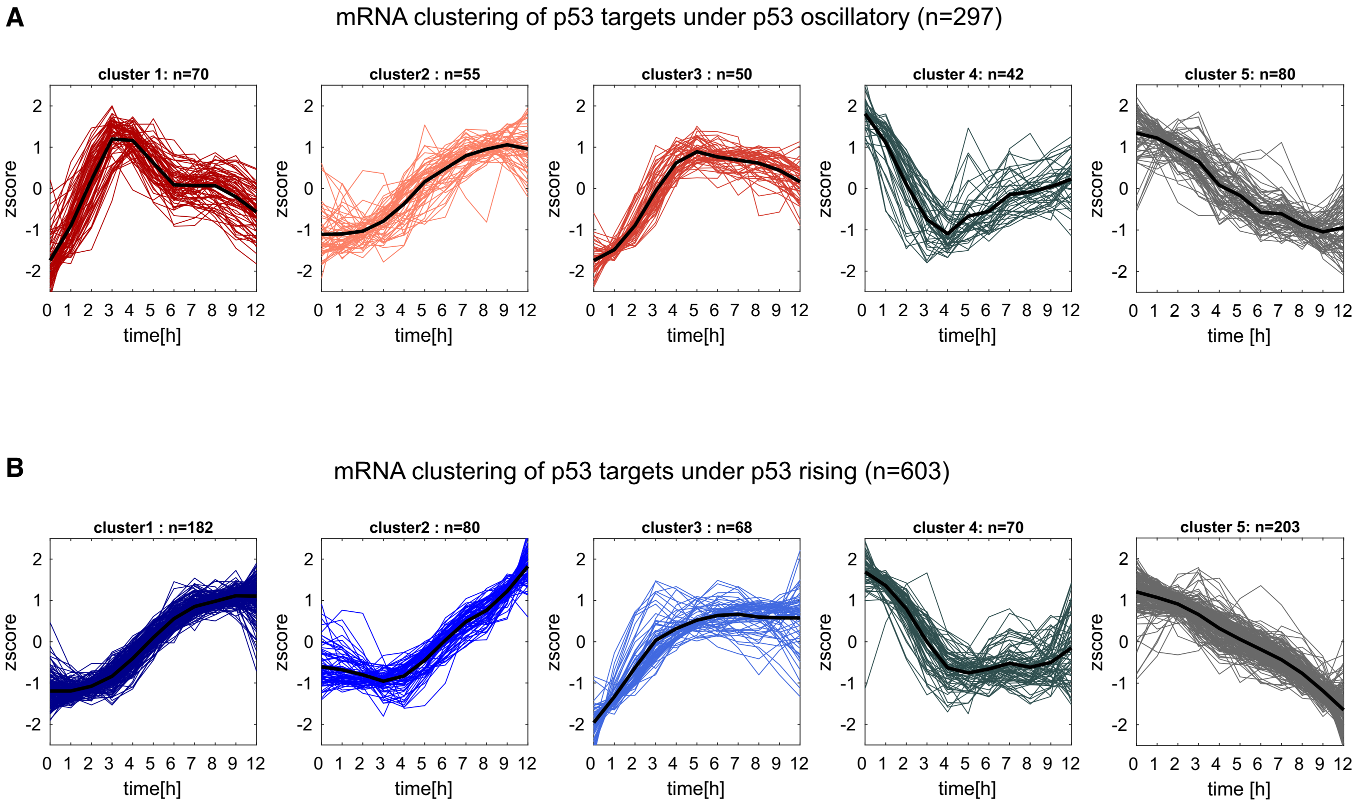

Figure EV1. Clustering of p53 mRNA targets under oscillating and pulsatile p53 expression.

A, B p53 target genes were clustered via fuzzy c-means clustering according to their mRNA expression profiles under (A) oscillatory or (B) rising p53 expression. Differential mRNA expression was defined as fold change > 1.5 and FDR < 0.2 (*t*-test, Benjamini–Hochberg corrected) based on two independent experiments. mRNAs were clustered based on their normalized time traces (*z* score) into five expression clusters under oscillatory and rising p53.

© 2022 The Authors

© 2022 The Authors
Molecular Systems Biology 18: e10588 | 2022 19 of 18

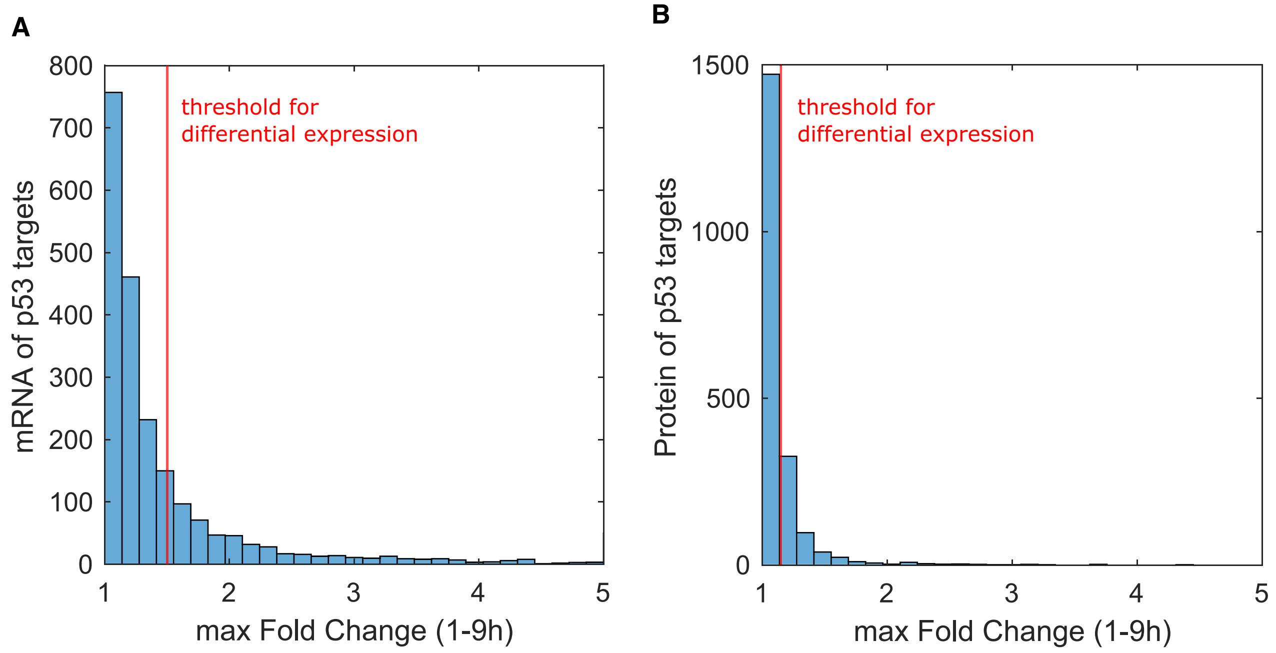

Figure EV2. Fold change in expression of mRNA and protein levels of p53 targets.

A, B Maximum fold-change (FC) distribution of mRNA and protein levels of p53 targets under both oscillatory and rising p53. Maximum fold change is calculated as the largest FC value from 1 to 9 h. Red vertical lines indicate fold-change threshold to consider an mRNA (FC > 1.5) or protein (FC > 1.15) differentially expressed.

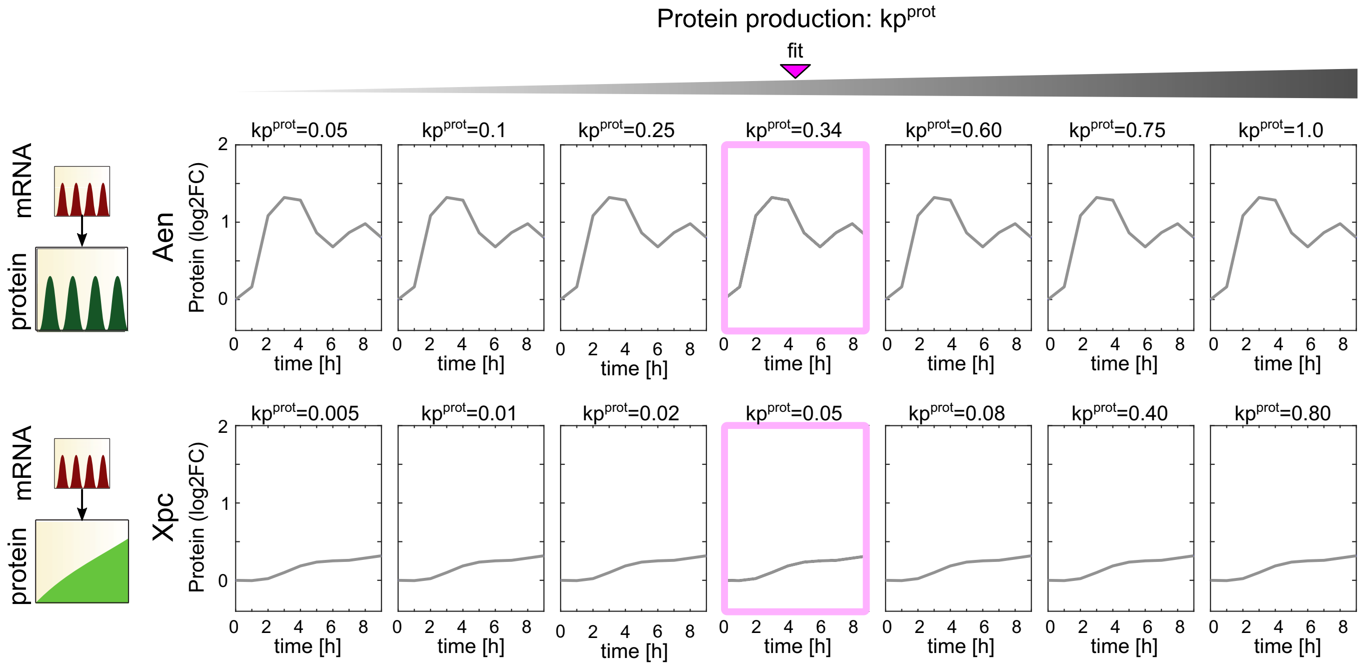

Figure EV3. Predicted protein expression trajectories of two representative p53 target genes for different values of kp^{prot}.

Protein expression trajectories for targets showing the mRNA and protein dynamics indicated at the left were modeled for the indicated values of kp^{prot} (with kd^{prot} fixed at the value determined in Fig 3C). Pink box shows the value of kp^{prot} that best fits the experimentally measured protein levels.

© 2022 The Authors

© 2022 The Authors

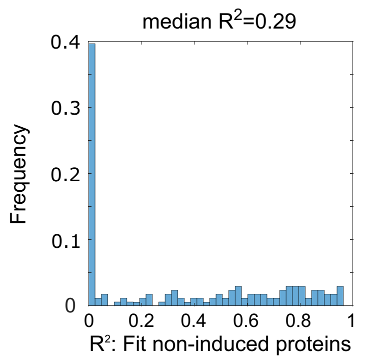

Figure EV4. Distribution of R^2 values for the fitting of expression of p53 target proteins not induced under oscillatory conditions.

All proteins that were detectable but not induced under oscillatory p53 were fit for kp^{prot} and kd^{prot} based on mass spectrometry data collected under oscillatory conditions. The distribution of R^2 values for these fits is shown.

◀ **Figure EV5. Expanded view of mechanisms decoding p53 dynamics.**

A Schematics showing relationship between dynamical mRNA and protein responses for oscillatory mRNA.

B Four targets with similar mRNA expression profiles—high mRNA degradation rate—but distinct protein profiles due to differences in protein degradation rates, activation thresholds (AT), or existence of cFFLs are shown.

C, D Venn diagrams showing exclusive or pervasive induction of mRNA and protein. Fifteen percent of mRNA (28/191 = 15%) and 20% (16/82 = 20%) of proteins show exclusive induction. A total of 109 of the 191 mRNAs in (C) failed to induce proteins under either p53 dynamic.

© 2022 The Authors Molecular Systems Biology 18: e10588 | 2022 21 of 18

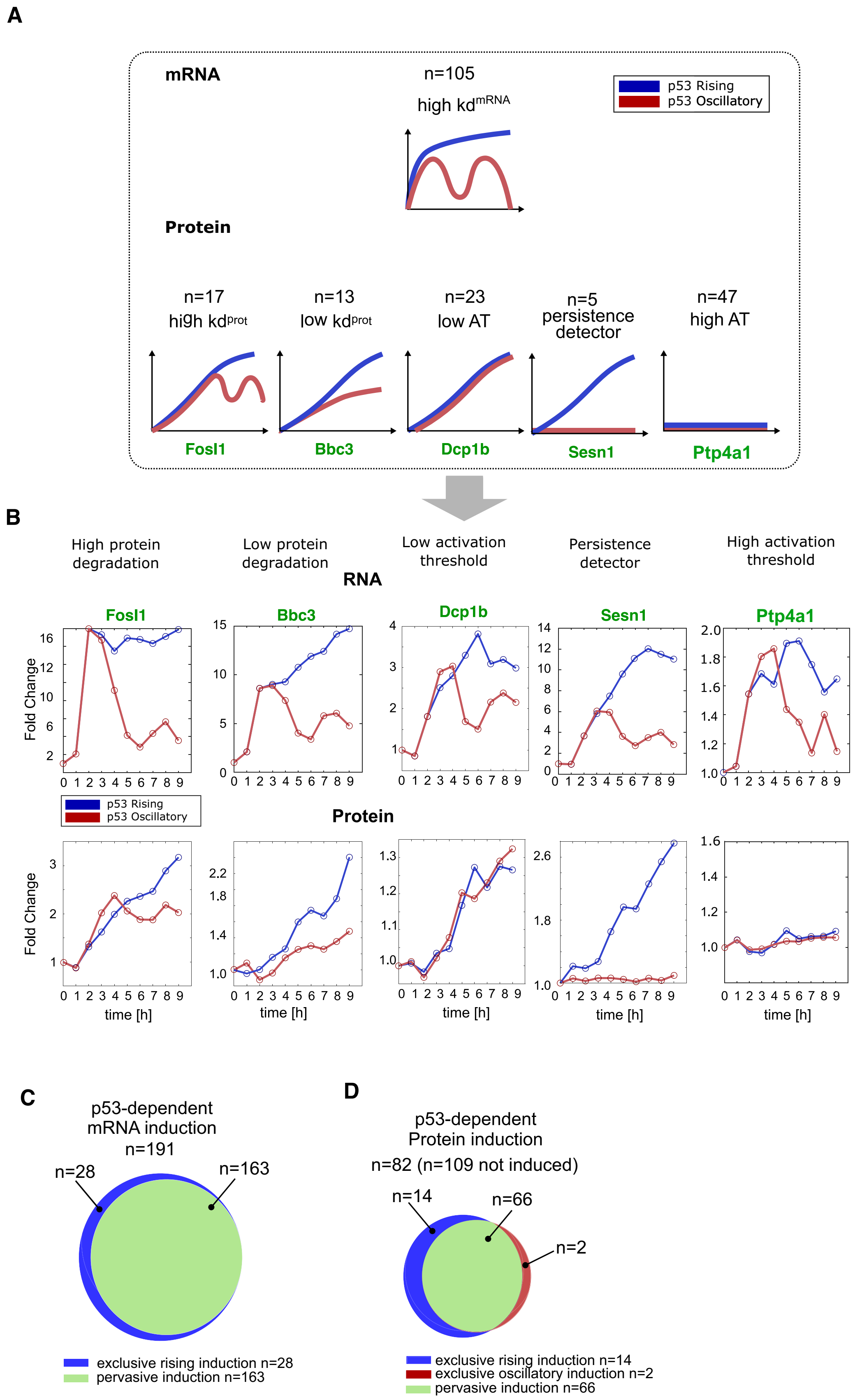

Figure EV5.

© 2022 The Authors
