# Supplementary material for: Time‐series transcriptomics and proteomics reveal alternative modes to decode p53 oscillations
Source: Mol Syst Biol. 2022 Mar 14;18(3):e10588. doi: 10.15252/msb.202110588 (PMC8919251; doi:10.15252/msb.202110588)
Supplement: Supplementary file 7 — Source Data for Figure 1 [file MSB-18-e10588-s007.pdf]

Source Data Figure1

All cropped Western Blots pertaining to Figure 1B-C

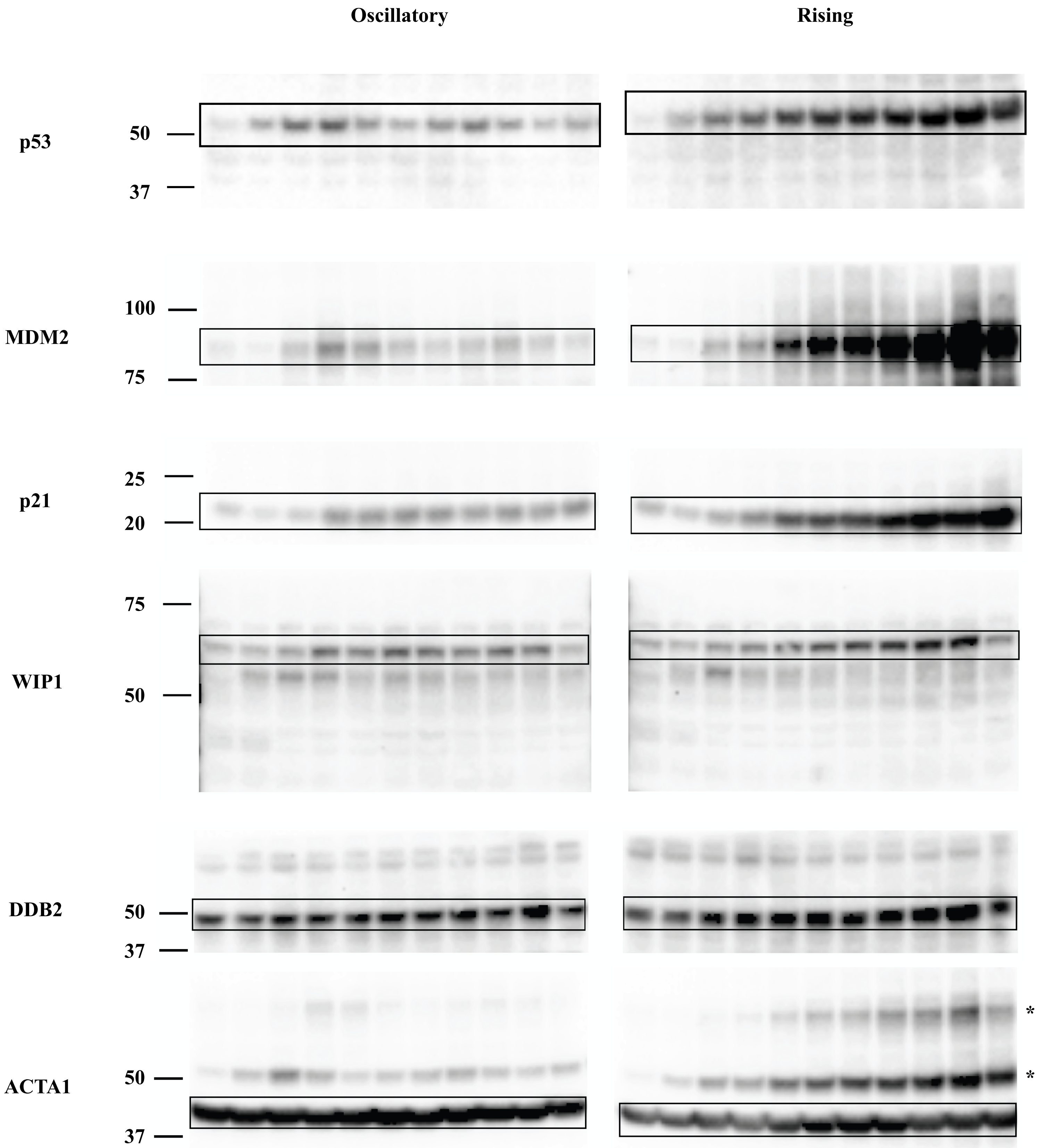

* = other proteins probed prior to Actin Western Blot

Source Data Figure 1: Uncropped scans of Western blot for p53 and select p53 targets under oscillatory and rising conditions. All ladder sizes/units are displayed in kDa. Stars denote proteins which have been probed prior to Actin.
