# Supplementary material for: Time‐series transcriptomics and proteomics reveal alternative modes to decode p53 oscillations
Source: Mol Syst Biol. 2022 Mar 14;18(3):e10588. doi: 10.15252/msb.202110588 (PMC8919251; doi:10.15252/msb.202110588)
Supplement: Supplementary file 8 [file MSB-18-e10588-s006.pdf]

molecular systems biology

Time-series transcriptomics and proteomics reveal alternative modes to decode p53 oscillations

Alba Jiménez, Dan Lu, Marian Kalocsay, Matthew J. Berberich, Petra Balbi, Ashwini Jambhekar, and Galit Lahav
DOI: 10.15252/msb.202110588

Corresponding author(s): Galit Lahav (galit@hms.harvard.edu) , Ashwini Jambhekar (Ashwini_Jambhekar@hms.harvard.edu)

Review Timeline:

Submission Date:	19th Jul 21
Editorial Decision:	16th Sep 21
Revision Received:	16th Dec 21
Editorial Decision:	9th Feb 22
Revision Received:	16th Feb 22
Accepted:	19th Feb 22

Editor: Jingyi Hou

Transaction Report:

(Note: With the exception of the correction of typographical or spelling errors that could be a source of ambiguity, letters and reports are not edited. Depending on transfer agreements, referee reports obtained elsewhere may or may not be included in this compilation. Referee reports are anonymous unless the Referee chooses to sign their reports.)

16th Sep 2021

Manuscript Number: MSB-2021-10588
Title: Time-series transcriptomics and proteomics reveal alternative modes to decode p53 oscillations
Author: Galit Lahav
Ashwini Jambhekar
Matthew J. Berberich
Dan Lu
Alba Jiménez
Marian Kalocsay
Petra Balbi

Dear Dr Lahav,

Thank you for submitting your work to Molecular Systems Biology. We have now heard back from two of the three reviewers who agreed to evaluate your manuscript. Unfortunately, after a series of reminders, we did not manage to obtain a report from Reviewer #3. In the interest of time, I prefer to make a decision now rather than further delaying the process. You will see from the comments below that Reviewers #1 and #2 find the manuscript to be of potential interest. They raise, however, several important points, which should be convincingly addressed in a revision of this work.

I think that the reviewers' recommendations are rather straightforward, and there is no need to reiterate their comments. Notably, the first and third concerns of Reviewer #2 regarding the molecular mechanisms and the generality of the presented findings in additional cell lines need to be addressed in order to enhance the conclusiveness and the level of biological insight provided by the study.

All other issues raised by the reviewers need to be satisfactorily addressed as well. As you may already know, our editorial policy allows in principle a single round of major revision, so it is essential to respond to the reviewers' comments that are as complete as possible.

On a more editorial level, we would ask you to address the following issues:

- Please provide a .docx formatted version of the manuscript text (including legends for main figures, EV figures and tables). Please make sure that the changes are highlighted to be clearly visible.

- Please provide individual production quality figure files as .eps, .tif, .jpg (one file per figure).

-Please provide a .docx formatted letter INCLUDING the reviewers' reports and your detailed point-by-point responses to their comments. As part of the EMBO Press transparent editorial process, the point-by-point response is part of the Review Process File (RPF), which will be published alongside your paper.

-Please note that all corresponding authors are required to supply an ORCID ID for their name upon submission of a revised manuscript.

-We replaced Supplementary Information with Expanded View (EV) Figures and Tables that are collapsible/expandable online (see examples in http://msb.embopress.org/content/11/6/812). A maximum of 5 EV Figures can be typeset. EV Figures should be cited as 'Figure EV1, Figure EV2" etc... in the text and their respective legends should be included in the main text after the legends of regular figures.

Additional Tables/Datasets should be labeled and referred to as Table EV1, Dataset EV1, etc. Legends have to be provided in a separate tab in case of .xls files. Alternatively, the legend can be supplied as a separate text file (README) and zipped together with the Table/Dataset file.

For the figures and tables that you do NOT wish to display as Expanded View figures, they should be bundled together with their legends in a single PDF file called *Appendix*, which should start with a short Table of Content. Each legend should be below the corresponding Figure/Table in the Appendix. Appendix figures and tables should be referred to in the main text as: "Appendix Figure S1, Appendix Figure S2, Appendix Table S1" etc. See detailed instructions regarding expanded view here: https://www.embopress.org/page/journal/17444292/authorguide#expandedview.

-Before submitting your revision, primary datasets (and computer code, where appropriate) produced in this study need to be deposited in an appropriate public database (see

https://www.embopress.org/page/journal/17444292/authorguide#dataavailability).

Please remember to provide a reviewer password if the datasets are not yet public.

The accession numbers and database should be listed in a formal "Data Availability" section (placed after Materials & Method) that follows the model below (see also https://www.embopress.org/page/journal/17444292/authorguide#dataavailability). Please note that the Data Availability Section is restricted to new primary data that are part of this study.

Data availability

The datasets (and computer code) produced in this study are available in the following databases:

- RNA-Seq data: Gene Expression Omnibus GSE46843 (https://www.ncbi.nlm.nih.gov/geo/query/acc.cgi?acc=GSE46843)
- [data type]: [name of the resource] [accession number/identifier/doi] ([URL or identifiers.org/DATABASE:ACCESSION])

*** Note - All links should resolve to a page where the data can be accessed. ***

- We would encourage you to include the source data for figure panels that show essential quantitative information. Additional information on source data and instruction on how to label the files are available at <
https://www.embopress.org/page/journal/17444292/authorguide#sourcedata
>.

- All Materials and Methods need to be described in the main text. We would encourage you to use 'Structured Methods', our new Materials and Methods format. According to this format, the Material and Methods section should include a Reagents and Tools Table (listing key reagents, experimental models, software and relevant equipment and including their sources and relevant identifiers) followed by a Methods and Protocols section in which we encourage the authors to describe their methods using a step-by-step protocol format with bullet points, to facilitate the adoption of the methodologies across labs. More information on how to adhere to this format as well as downloadable templates (.doc or .xls) for the Reagents and Tools Table can be found in our author guidelines: < https://www.embopress.org/page/journal/17444292/authorguide#researcharticleguide>. An example of a Method paper with Structured Methods can be found here: .

-Regarding data quantification:
Please ensure to specify the name of the statistical test used to generate error bars and P values, the number (n) of independent experiments (please specify technical or biological replicates) underlying each data point and the test used to calculate p-values in each figure legend. Discussion of statistical methodology can be reported in the materials and methods section, but figure legends should contain a basic description of n, P and the test applied.
Graphs must include a description of the bars and the error bars (s.d., s.e.m.).
Please also include scale bars in all microscopy images.

- Please provide a "standfirst text" summarizing the study in one or two sentences (approximately 250 characters, including space), three to four "bullet points" highlighting the main findings and a "synopsis image" (550px width and 300-600 px height, PNG format) to highlight the paper on our homepage.
Here are a couple of examples:
https://www.embopress.org/doi/10.15252/msb.20199356
https://www.embopress.org/doi/10.15252/msb.20209475
https://www.embopress.org/doi/10.15252/msb.209495

When you resubmit your manuscript, please download our CHECKLIST (http://bit.ly/EMBOPressAuthorChecklist) and include the completed form in your submission.
Please note that the Author Checklist will be published alongside the paper as part of the transparent process (https://www.embopress.org/page/journal/17444292/authorguide#transparentprocess).

If you feel you can satisfactorily deal with these points and those listed by the referees, you may wish to submit a revised version of your manuscript. Please attach a covering letter giving details of the way in which you have handled each of the points raised by the referees. A revised manuscript will be once again subject to review and you probably understand that we can give you no guarantee at this stage that the eventual outcome will be favorable.

I look forward to seeing your revised manuscript.

Sincerely,
Jingyi

Jingyi Hou

Editor
Molecular Systems Biology

If you do choose to resubmit, please click on the link below to submit the revision online *within 90 days*.

https://msb.msubmit.net/cgi-bin/main.plex

IMPORTANT: When you send your revision, we will require the following items:
1. the manuscript text in LaTeX, RTF or MS Word format
2. a letter with a detailed description of the changes made in response to the referees. Please specify clearly the exact places in the text (pages and paragraphs) where each change has been made in response to each specific comment given
3. three to four 'bullet points' highlighting the main findings of your study
4. a short 'blurb' text summarizing in two sentences the study (max. 250 characters)
5. a 'thumbnail image' (550px width and max 400px height, Illustrator, PowerPoint or jpeg format), which can be used as 'visual title' for the synopsis section of your paper.
6. Please include an author contributions statement after the Acknowledgements section (see https://www.embopress.org/page/journal/17444292/authorguide)
7. Please complete the CHECKLIST available at (https://bit.ly/EMBOPressAuthorChecklist).
Please note that the Author Checklist will be published alongside the paper as part of the transparent process (https://www.embopress.org/page/journal/17444292/authorguide#transparentprocess).
8. Please note that corresponding authors are required to supply an ORCID ID for their name upon submission of a revised manuscript (EMBO Press signed a joint statement to encourage ORCID adoption).
(https://www.embopress.org/page/journal/17444292/authorguide#editorialprocess)

Currently, our records indicate that there is no ORCID associated with your account.

Please click the link below to provide an ORCID:
Link Not Available

The system will prompt you to fill in your funding and payment information. This will allow Wiley to send you a quote for the article processing charge (APC) in case of acceptance. This quote takes into account any reduction or fee waivers that you may be eligible for. Authors do not need to pay any fees before their manuscript is accepted and transferred to the publisher.

As a matter of course, please make sure that you have correctly followed the instructions for authors as given on the submission website.

*** PLEASE NOTE *** As part of the EMBO Press transparent editorial process initiative (see our Editorial at https://dx.doi.org/10.1038/msb.2010.72), Molecular Systems Biology publishes online a Review Process File with each accepted manuscripts. This file will be published in conjunction with your paper and will include the anonymous referee reports, your point-by-point response and all pertinent correspondence relating to the manuscript. If you do NOT want this File to be published, please inform the editorial office at msb@embo.org within 14 days upon receipt of the present letter.

Reviewer #1:

In this article, Alba Jimenez and colleagues compare two modalities of p53 dynamics, oscillatory and sustained, in a breast cancer cell line MCF7. The authors use x-radiation to induce oscillatory p53 dynamics that becomes sustained in the presence of the MDM2 inhibitor Nutlin-3a according to a protocol established in the same lab. After inducing these two dynamic profiles, the authors fixed the cells at different time points from the induction, and then they analyzed the transcriptome and the proteome.
Using this method, the authors could analyze how mRNA and protein levels follow the dynamics of the transcription factor p53. By clustering of transcript and protein dynamics the authors observed different classes, from oscillatory mRNA and protein expression, to flat or continuously rising. The p53 oscillatory modality gives the highest diversity of dynamics outputs of mRNA and proteins. On the contrary, the sustained p53 dynamics determines simpler mRNA and protein dynamics. To better understand these differences, the authors use computational modeling, discovering the minimal regulatory circuits able to explain the observed mRNA and protein expression dynamics in response to the two modalities of p53 dynamics. By modifying in silico parameters such as mRNA and protein degradation, the authors could reproduce some observed classes of mRNA and protein dynamics.
This article represents an important resource to understand how the dynamics of a transcription factor is transcribed and

translated into the dynamics of its mRNA and protein targets. The experiments are carefully executed and analyzed, and the mathematical modelling gives an intuition about the logic of the regulatory network responsible for the different dynamic patterns observed in the experiment.
In our opinion, the article is appropriate for Molecular Systems Biology. However, we feel that there are still some unclear points that should be addressed before publication.

Major points:
1) In the Western Blots in figure 1 the authors seem to use different exposure to p53 and MDM2 in oscillatory vs sustained. According to the experimental protocol, at 2 hours the p53 and MDM2 expression levels should be the same in the two conditions and then stay high in the case of the sustained p53 expression. In our opinion, the authors should provide quantification or keep the same exposure for both the conditions for a fair comparison.
2) What the authors call "sustained" p53 expression is not sustained according to our interpretation, rather continuously rising. This is very visible in the western blot 1C and in contrast with the cartoons depicted in the paper. This has important consequences for the interpretation of the results, because the "sustained" condition not only abrogates the oscillations but also leads to levels of p53 expression that are much higher than the peaks of the oscillation. The authors should better describe this "continuous rising" dynamics. Moreover, this reviewer would like to know if this is taken into account in the computational model and what would be the consequences of that.
3) In figure 2B the authors show in some classes only 3 proteins per class. Is it the entire population of the class? It would be helpful to add population counts in all panels of Figure 2 just as it's been done in Figure EV1.
4) In the figure 1E the authors show the comparison between Mass Spec and WB from some selected proteins in the case of oscillatory p53. The authors should show the same for the case of sustained p53.
5) Figure 4C would benefit from a better layout. For example, making clear that the protein dynamics are part of the rows, by putting the cartons in the frames or in a simple table layout. Regarding the same figure, we had problems to understand why "n = 0" in row 4 column 3 has no asterisk, it seems like this scenario would be difficult to achieve without additional complex regulation. Further, it is not easy to understand that the category "i" mentioned at line 273 corresponds to regulation from the p53 dynamics to mRNA dynamics and not the regulation on the protein level from mRNA which is stated as "simple regulation" in figure 4C.

Minor points:
1) The Western Blots in figure 1 appear saturated and the saturated part appears at a low resolution. The authors should provide the original images for comparison.
2) line 391 the authors should clarify why in their opinion the second alternative is less likely

Textual:
1) At line 203 a space is missing
2) At line 485 a closing bracket is missing
3) The sentence at line 218 is correct, but quite confusing

Reviewer #2:

The p53 tumor suppressor mediates the majority of its myriad effects via differential regulation of gene expression. Previous studies have suggested that p53-dependent gene expression occurs in distinct manners depending upon whether p53 oscillates or is sustained in its expression. In this manuscript, the authors perform a comprehensive and thorough analysis of the transcriptomics and proteomics that arise depending upon whether p53 expression oscillates or is sustained. Several models are proposed to characterize these various modes of p53 target expression at both the RNA and protein levels.

Understanding how p53 controls gene expression is a critically important area of study for two main reasons. First, p53 represents an excellent model system in which to explore mechanisms by which oscillating versus sustained signals influence downstream target expression. Second, as p53 is a critically important player in human cancer, understanding its mechanisms of action will provide insights into its role in tumorigenesis. Thus, this study is certainly within the scope of Molecular Systems Biology. There are however four main concerns which make the manuscript not suitable for publication at this time.

First, the authors provide a comprehensive catalogue of gene and protein expression in MCF7 cells with various treatments. While this has some utility being made available to the field, the authors do not provide sufficient new mechanistic insights into the observed effects. Models are proposed but these tend to be expected and do not provide any underlying molecular explanations for the findings. Thus, the study is largely descriptive in nature. There is speculation about modeling withot sufficient experimental validation.

Second, the comparison of oscillating versus sustained p53 expression is exciting to explore. Yet, there may be other things

going on here beyond this. The authors compare radiation treatment alone with that which also includes use of the Mdm2 inhibitor nutlin. It is reasonable to speculate that findings may say more about the effect of nutlin than they do about oscillating versus sustained p53. This needs to be addressed more clearly in the manuscript.

Third, the studies are performed with a single cancer cell line which certainly has sustained multiple genetic alterations. It is unclear how universal the findings may be either for other tumor cell lines or for normal cells.

Fourth, the statistical cutoffs that are used (FDR<.2, fold>1.5) are rather generous and yet the authors identify small numbers of genes. This is surprising in light of the the numbers of p53 target genes that are identified in multiple other studies.

Dear Dr. Hou,

Thank you for agreeing to consider a revised version of our manuscript "Time-series transcriptomics and proteomics reveal alternative modes to decode p53 oscillations". We have addressed the reviewers' comments as detailed below. We added new supplementary figures of experimental data and analysis and revised the text, figures, table and figure legends to clarify points that the referees found confusing or unclear.

We thank you and the reviewers for the constructive and detailed input, which has helped us improve the paper, and many thanks for your comments and efforts with this revision.

Sincerely,
Galit

Reviewer #1:

This article represents an important resource to understand how the dynamics of a transcription factor is transcribed and translated into the dynamics of its mRNA and protein targets. The experiments are carefully executed and analyzed, and the mathematical modelling gives an intuition about the logic of the regulatory network responsible for the different dynamic patterns observed in the experiment. In our opinion, the article is appropriate for Molecular Systems Biology. However, we feel that there are still some unclear points that should be addressed before publication.

We thank the reviewer for the positive comments and for appreciating the contributions of our work to the field.

Major points:

1) In the Western Blots in figure 1 the authors seem to use different exposure to p53 and MDM2 in oscillatory vs sustained. According to the experimental protocol, at 2 hours the p53 and MDM2 expression levels should be the same in the two conditions and then stay high in the case of the sustained p53 expression. In our opinion, the authors should provide quantification or keep the same exposure for both the conditions for a fair comparison.

The amount of p53 at 2 hours is indeed equal under the two different dynamical conditions and we apologize if the different exposed blots made it difficult to compare and appreciate. We have now provided blots with the same exposure time for both oscillatory and sustained (now termed 'rising') p53 (updated **Figure 1B-C**).

2) What the authors call "sustained" p53 expression is not sustained according to our interpretation, rather continuously rising. This is very visible in the western blot 1C and in contrast with the cartoons depicted in the paper. This has important consequences for the interpretation of the results, because the "sustained" condition not only abrogates the oscillations but also leads to levels of p53 expression that are much higher than the peaks of the oscillation. The authors should better describe this "continuous rising" dynamics. Moreover, this reviewer would like to know if this is taken into account in the computational model and what would be the consequences of that.

We appreciate the reviewer's careful analysis of the data, and we agree that p53 levels continue to rise under the "sustained" regime. To address the reviewer's comment and better capture the observed dynamics we have changed the labels in the text and figures from "sustained' to "rising" and provided schematics that show rising rather than plateauing p53 levels.

With regards to the potential impact on our computational models: all computational models in the paper were conducted using real experimental p53 values from mass spectrometry measurements. Our choice of descriptor for the p53 dynamics induced by Nutlin-3 ("sustained" vs "rising") therefore does not affect the outcomes of the models. We have now clarified in the text (lines 198 & 261) that the modeling is based on empirical measurements of p53 levels by mass spectrometry.

3) In figure 2B the authors show in some classes only 3 proteins per class. Is it the entire population of the class? It would be helpful to add population counts in all panels of Figure 2 just as it's been done in Figure EV1.

We agree with the reviewer that additional information about the dataset size at each stage would be helpful for readers. We have added a new figure in the appendix (Appendix new **Figure S1**) that describes the filters applied at each stage in **Figures 2, 3 and 4**, and the number of p53 targets that pass each filter.

4) In the figure 1E the authors show the comparison between Mass Spec and WB from some selected proteins in the case of oscillatory p53. The authors should show the same for the case of sustained p53.

We agree with the reviewer, and have now provided additional graphs showing quantification by mass spec and western blot of select p53 target genes under "sustained" (now called "rising") dynamics (new **Figure 1 F, G**).

5) Figure 4C would benefit from a better layout. For example, making clear that the protein dynamics are part of the rows, by putting the cartons in the frames or in a simple table layout.

Regarding the same figure, we had problems to understand why "n = 0" in row 4 column 3 has no asterisk, it seems like this scenario would be difficult to achieve without additional complex regulation. Further, it is not easy to understand that the category "i" mentioned at line 273 corresponds to regulation from the p53 dynamics to mRNA dynamics and not the regulation on the protein level from mRNA which is stated as "simple regulation" in figure 4C.

We appreciate the reviewer's suggestions for improving the clarity of this figure. We rearranged **Figure 4C** in the form of a table and added two colors to distinguish transcriptional mechanisms (pink) from translational ones (green). This new presentation should create a more comprehensive and clearer layout.

We also thank the reviewer for the comment on the asterisk. All categories with n=0, including category (i), can indeed only be explained through additional complex regulation. We have marked these cases with an asterisk and explained in the figure legend and in the text (lines 271-272) that these can be explained only through complex regulation.

Minor points:

1) The Western Blots in figure 1 appear saturated and the saturated part appears at a low resolution. The authors should provide the original images for comparison.

New western blot panels (updated **Figure 1 B, C**) are now provided in response to this point and Major Point 1, as well as the quantitation of select targets under rising p53 dynamics (new **Figure 1F-G**).

2) line 391 the authors should clarify why in their opinion the second alternative is less likely

We hypothesize that cellular outcomes, particularly death under rising dynamics, are not driven by exclusive induction of specific genes because the key apoptosis drivers are induced under both dynamical conditions. We have edited the sentence to better communicate this idea (lines 405-411).

Textual:
1) At line 203 a space is missing
2) At line 485 a closing bracket is missing

We have corrected the text at these lines.

3) The sentence at line 218 is correct, but quite confusing.

We separated the ideas in this sentence into 2 sentences to better express our reasoning.

Reviewer #2:

Understanding how p53 controls gene expression is a critically important area of study for two main reasons. First, p53 represents an excellent model system in which to explore mechanisms by which oscillating versus sustained signals influence downstream target expression. Second, as p53 is a critically important player in human cancer, understanding its mechanisms of action will provide insights into its role in tumorigenesis. Thus, this study is certainly within the scope of Molecular Systems Biology. There are however four main concerns which make the manuscript not suitable for publication at this time.

We thank the reviewer for their support of our work.

First, the authors provide a comprehensive catalogue of gene and protein expression in MCF7 cells with various treatments. While this has some utility being made available to the field, the authors do not provide sufficient new mechanistic insights into the observed effects. Models are proposed but these tend to be expected and do not provide any underlying molecular explanations for the findings. Thus, the study is largely descriptive in nature. There is speculation about modeling without sufficient experimental validation.

Our study represents an important step forward in understanding how the dynamics of a transcription factor is transcribed and translated into the dynamics of its mRNA and protein targets globally, and offer the first systematic overview about the simplest regulatory motifs responsible for the different dynamic patterns observed in the experiment. We expect that some of the paradigms we propose will hold in other systems, e.g. that of multiple network motifs operating downstream of a single transcription factor, or of a multi-functional transcription factor exhibiting low specificity of target pathway selection. This, in our opinion, is not less novel or less important than identifying specific molecular mechanisms.

We do agree that identifying the specific molecular factors in the models will be an interesting follow up. However, such efforts are beyond the scope of a major revision. Importantly, our approach of looking at the dynamics of a key transcription factor together with the dynamics of *all* its mRNA and protein targets was essential for guiding us and others where to look for such potential molecular mechanisms. In the revised discussion we have proposed a few molecular mechanisms based on the literature, and have now better linked these mechanisms to our models (lines 435-451). We have also provided a new paragraph in the discussion (lines 453-

464) detailing the experimental observations in the literature on which our choice of minimal networks motifs is based.

Second, the comparison of oscillating versus sustained p53 expression is exciting to explore. Yet, there may be other things going on here beyond this. The authors compare radiation treatment alone with that which also includes use of the Mdm2 inhibitor nutlin. It is reasonable to speculate that findings may say more about the effect of nutlin than they do about oscillating versus sustained p53. This needs to be addressed more clearly in the manuscript.

The reviewer is correct to inquire about potential off-target effects of nutlin-3a. This molecule was developed in 2004 and has been extensively studied since. Early microarray studies (Tovar et al. 2006) as well as more recent genome-wide expression studies (Allen et al. 2014) have shown that nutlin has minimal to no effects on gene expression in cells lacking p53. In our work, our maximal nutlin dosage (4 μM) is less than half of those used in both studies mentioned above (10 μM). These studies suggest that the major effects of nutlin occur through its modulation of p53 dynamics. Nutlin and its derivatives are currently in clinical trials aimed at inducing cancer cell death by manipulating p53 dynamics. We have now cited these studies (lines 110-112) to justify our choice of treatment.

Third, the studies are performed with a single cancer cell line which certainly has sustained multiple genetic alterations. It is unclear how universal the findings may be either for other tumor cell lines or for normal cells.

The main findings of our study required precise and extensive *global* dynamical measurements including time-series mRNA sequencing and time-series mass spec experiments. Repeating these in another cell line is extremely costly and time intensive, and therefore beyond the scope of a major revision. We expect that the "big picture" ideas-- such as diversity of gene expression patterns and use of different network motifs to regulate target expression—will recur in other systems. However, these cannot be predicted by measuring and validating the behavior of a few selected genes across cell lines. In fact, we expect that behaviors of some specific genes may differ, which would neither invalidate nor augment our results. That being said, we note that similar expression patterns were reported in the human non-cancerous RPE cells for almost all the target genes that were tested at Hanson et al. 2019 (see below). We now noted this similarity in the text, as well as raised the broader issue of conservation of mechanisms as an area worthy of future exploration (lines 227-232 & 377-382).

Data from Figure S4 Hanson et al. 2009

Dynamic protein expression of 9 p53 targets in hTERT-RPE1 cells. Acquired through Western Blot.

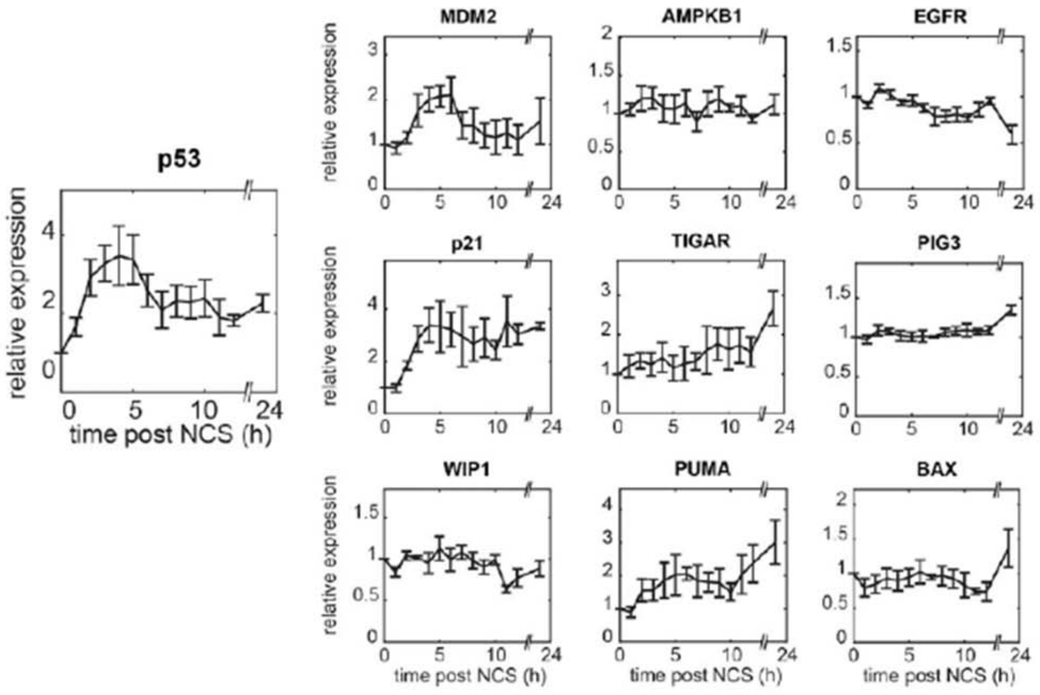

Figure S4. **p53 target protein expression dynamics in different cell lines.** Related to Fig. 4. Western blot analysis of the expression of p53 and the indicated downstream targets in response to 400 ng/ml NCS treatment in hTERT-RPE1 cells

Data from our study.

Dynamic protein expression of the corresponding targets in MCF7 cells. Acquired through Mass Spectometry.

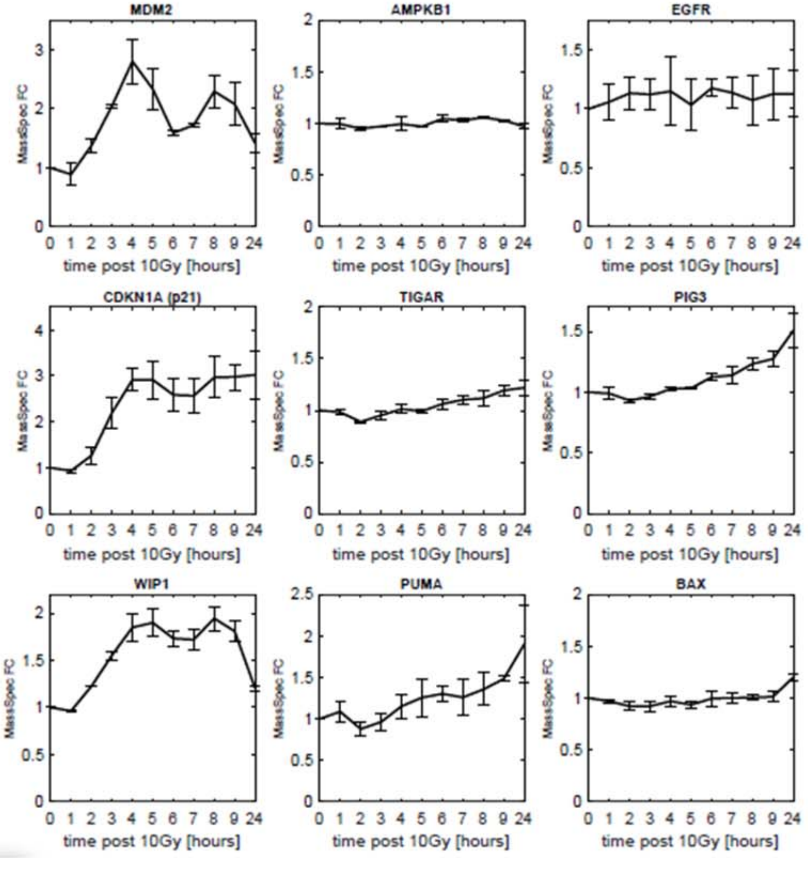

Fourth, the statistical cutoffs that are used (FDR<.2, fold>1.5) are rather generous and yet the authors identify small numbers of genes. This is surprising in light of the numbers of p53 target genes that are identified in multiple other studies.

We thank the reviewer for prompting us to describe our data processing with greater transparency. Our data were processed through multiple filters in order to ensure reproducibility between biological replicates and to make direct comparisons between RNA and protein expression under different p53 dynamics. We have now added a new figure in the appendix that describes the size of each dataset and the number of p53 targets that pass each filter (Appendix New **Figure S1**).

The reviewer might find it helpful to know that our gene sets are similar in size to those identified in the literature using similar filtering strategies. For example, in 2020 Moyer et al. analyzed genes that were differentially expressed in various tissues following p53 activation by nutlin-3a, and the number ranged from 61 (intestine) to 747 (pancreas). Upon filtering for direct p53 targets based on ChIP-seq data and for genes that were upregulated, these numbers were ranged from 20 (ovary) to 186 (pancreas). In another study from 2013, Kenzelmann Broz et al. found that after DNA damage, p53 was bound to more than 3000 genes by ChIP-Seq, of which only 365 were induced and 67 were repressed. In our own dataset, after applying similar filters for upregulation and direct binding by p53, we identified 175 induced target genes for oscillatory p53 and 330 for sustained (now termed 'rising') (Appendix new **Figure S1**). These values are within the ranges reported in the studies above. We now specifically mention in the text (lines 154-156) that the size of our p53 target gene sets is comparable to what others have previously reported, and added the corresponding references.

9th Feb 2022

Manuscript Number: MSB-2021-10588R
Title: Time-series transcriptomics and proteomics reveal alternative modes to decode p53 oscillations
Author: Alba Jiménez
Dan Lu
Marian Kalocsay
Matthew J. Berberich
Petra Balbi
Ashwini Jambhekar
Galit Lahav

Thank you for sending us your revised manuscript. We have now heard back from one of the two reviewers who agreed to evaluate your study. Unfortunately, after a series of reminders, we did not obtain a report from Reviewer #2. In the interest of time, I prefer to make a decision now rather than further delaying the process. As you will see below, the reviewer is satisfied with the modifications made and thinks that the study is now suitable for publication.

Before we can formally accept your manuscript, we would ask you to address the following editorial-level issues:

As a matter of course, please make sure that you have correctly followed the instructions for authors as given on the submission website.

Thank you for submitting this paper to Molecular Systems Biology. I look forward to receiving the revised version soon.

Kind regards,
Jingyi

Jingyi Hou
Editor
Molecular Systems Biology

If you do choose to resubmit, please click on the link below to submit the revision online before 11th Mar 2022.

https://msb.msubmit.net/cgi-bin/main.plex

IMPORTANT: When you send your revision, we will require the following items:

1. the manuscript text in LaTeX, RTF or MS Word format
2. a letter with a detailed description of the changes made in response to the referees. Please specify clearly the exact places in the text (pages and paragraphs) where each change has been made in response to each specific comment given
3. three to four 'bullet points' highlighting the main findings of your study
4. a short 'blurb' text summarizing in two sentences the study (max. 250 characters)
5. a 'thumbnail image' (550px width and max 400px height, Illustrator, PowerPoint or jpeg format), which can be used as 'visual title' for the synopsis section of your paper.
6. Please include an author contributions statement after the Acknowledgements section (see https://www.embopress.org/page/journal/17444292/authorguide#manuscriptpreparation)
7. Please complete the CHECKLIST available at (https://bit.ly/EMBOPressAuthorChecklist).
Please note that the Author Checklist will be published alongside the paper as part of the transparent process (https://www.embopress.org/page/journal/17444292/authorguide#transparentprocess).
8. When assembling figures, please refer to our figure preparation guideline in order to ensure proper formatting and readability in print as well as on screen:
https://bit.ly/EMBOPressFigurePreparationGuideline
See also figure legend guidelines: https://www.embopress.org/page/journal/17444292/authorguide#figureformat
9. Please note that corresponding authors are required to supply an ORCID ID for their name upon submission of a revised manuscript (EMBO Press signed a joint statement to encourage ORCID adoption).
(https://www.embopress.org/page/journal/17444292/authorguide#editorialprocess)
Currently, our records indicate that the ORCID for your account is 0000-0003-4758-6427.

Please click the link below to modify this ORCID:
Link Not Available

The system will prompt you to fill in your funding and payment information. This will allow Wiley to send you a quote for the article processing charge (APC) in case of acceptance. This quote takes into account any reduction or fee waivers that you may be eligible for. Authors do not need to pay any fees before their manuscript is accepted and transferred to the publisher.

As a matter of course, please make sure that you have correctly followed the instructions for authors as given on the submission website.

*** PLEASE NOTE *** As part of the EMBO Press transparent editorial process initiative (see our Editorial at https://dx.doi.org/10.1038/msb.2010.72 , Molecular Systems Biology will publish online a Review Process File to accompany accepted manuscripts. When preparing your letter of response, please be aware that in the event of acceptance, your cover letter/point-by-point document will be included as part of this File, which will be available to the scientific community. More information about this initiative is available in our Instructions to Authors. If you have any questions about this initiative, please contact the editorial office (msb@embo.org).

Reviewer #1:

In the revised version of their article, Alba Jimenez and colleagues answered to my concerns and those of the other reviewer. I appreciate they efforts to increase the clarity of the manuscript. In particular, changing of definition from "sustained" to "rising" better describes the observed data. I don't have any further concerns.
I think that this paper is an important resource in the field of signaling dynamics.

Dear Dr. Hou,

Thank you for your guidance in preparing this manuscript. We have made all of the requested changes, and we hope this manuscript is now suitable for publication.

Galit Lahav

19th Feb 2022
Manuscript number: MSB-2021-10588RR
Title: Time-series transcriptomics and proteomics reveal alternative modes to decode p53 oscillations

Dear Dr Lahav,

Thank you again for sending us your revised manuscript. We are now satisfied with the modifications made and I am pleased to inform you that your paper has been accepted for publication.

*** PLEASE NOTE *** As part of the EMBO Publications transparent editorial process initiative (see our Editorial at https://dx.doi.org/10.1038/msb.2010.72), Molecular Systems Biology publishes online a Review Process File with each accepted manuscripts. This file will be published in conjunction with your paper and will include the anonymous referee reports, your point- by-point response and all pertinent correspondence relating to the manuscript. If you do NOT want this File to be published, please inform the editorial office at msb@embo.org within 14 days upon receipt of the present letter.

Should you be planning a Press Release on your article, please get in contact with msb@wiley.com as early as possible, in order to coordinate publication and release dates.

LICENSE AND PAYMENT:
All articles published in Molecular Systems Biology are fully open access: immediately and freely available to read, download and share.

Molecular Systems Biology charges an article processing charge (APC) to cover the publication costs. You, as the corresponding author for this manuscript, should have already received a quote with the article processing fee separately. Please let us know in case this quote has not been received.

Once your article is at Wiley for editorial production you will receive an email from Wiley's Author Services system, which will ask you to log in and will present you with the publication license form for completion. Within the same system the publication fee can be paid by credit card, an invoice or pro forma can be requested.

Payment of the publication charge and the signed Open Access Agreement form must be received before the article can be published online.

Molecular Systems Biology articles are published under the Creative Commons licence CC BY, which facilitates the sharing of scientific information by reducing legal barriers, while mandating attribution of the source in accordance to standard scholarly practice.

Proofs will be forwarded to you within the next 2-3 weeks.

Thank you very much for submitting your work to Molecular Systems Biology.

Sincerely,

Jingyi Hou
Editor
Molecular Systems Biology

EMBO PRESS

YOU MUST COMPLETE ALL CELLS WITH A PINK BACKGROUND

PLEASE NOTE THAT THIS CHECKLIST WILL BE PUBLISHED ALONGSIDE YOUR PAPER

Corresponding Author Name: Galit Lahav, Ashwini Jambhekar

Journal Submitted to: Molecular Systems Biology
Manuscript Number: MSB-2021-10588

Reporting Checklist For Life Sciences Articles (Rev. June 2017)

This checklist is used to ensure good reporting standards and to improve the reproducibility of published results. These guidelines are consistent with the Principles and Guidelines for Reporting Preclinical Research issued by the NIH in 2014. Please follow the journal's authorship guidelines in preparing your manuscript.

A- Figures

1. Data

The data shown in figures should satisfy the following conditions:

- the data were obtained and processed according to the field's best practice and are presented to reflect the results of the experiments in an accurate and unbiased manner.
- figure panels include only data points, measurements or observations that can be compared to each other in a scientifically meaningful way.
- graphs include clearly labeled error bars for independent experiments and sample sizes. Unless justified, error bars should not be shown for technical replicates.
- if n< 5, the individual data points from each experiment should be plotted and any statistical test employed should be justified
- Source Data should be included to report the data underlying graphs. Please follow the guidelines set out in the author ship guidelines on Data Presentation.

2. Captions

Each figure caption should contain the following information, for each panel where they are relevant:

- a specification of the experimental system investigated (eg cell line, species name).
- the assay(s) and method(s) used to carry out the reported observations and measurements
- an explicit mention of the biological and chemical entity(ies) that are being measured.
- an explicit mention of the biological and chemical entity(ies) that are altered/varied/perturbed in a controlled manner.

- the exact sample size (n) for each experimental group/condition, given as a number, not a range;
- a description of the sample collection allowing the reader to understand whether the samples represent technical or biological replicates (including how many animals, litters, cultures, etc.).
- a statement of how many times the experiment shown was independently replicated in the laboratory.
- definitions of statistical methods and measures:
  - common tests, such as t-test (please specify whether paired vs. unpaired), simple χ2 tests, Wilcoxon and Mann-Whitney tests, can be unambiguously identified by name only, but more complex techniques should be described in the methods section;
  - are tests one-sided or two-sided?
  - are there adjustments for multiple comparisons?
  - exact statistical test results, e.g., P values = x but not P values < x;
  - definition of 'center values' as median or average;
  - definition of error bars as s.d. or s.e.m.

Any descriptions too long for the figure legend should be included in the methods section and/or with the source data.

USEFUL LINKS FOR COMPLETING THIS FORM

http://www.antibodypedia.com
http://1degreebio.org
http://www.equator-network.org/reporting-guidelines/improving-bioscience-research-repor
http://grants.nih.gov/grants/olaw/olaw.htm
http://www.mrc.ac.uk/Ourresearch/Ethicsresearchguidance/Useofanimals/index.htm
http://ClinicalTrials.gov
http://www.consort-statement.org
http://www.consort-statement.org/checklists/view/32-consort/66-title
http://www.equator-network.org/reporting-guidelines/reporting-recommendations-for-tumo
http://datadryad.org
http://figshare.com
http://www.ncbi.nlm.nih.gov/gap
http://www.ebi.ac.uk/ega
http://biomodels.net/
http://biomodels.net/miriam/
http://jjj.biochem.sun.ac.za
https://osp.od.nih.gov/biosafety-biosecurity-and-emerging-biotechnology/
http://www.selectagents.gov/

In the pink boxes below, please ensure that the answers to the following questions are reported in the manuscript itself. Every question should be answered. If the question is not relevant to your research, please write NA (non applicable). We encourage you to include a specific subsection in the methods section for statistics, reagents, animal models and human subjects.

B- Statistics and general methods

Please fill out these boxes (Do not worry if you cannot see all your text once you press return)

1.a. How was the sample size chosen to ensure adequate power to detect a pre-specified effect size?	Two biological replicates at each time point, both for RNA-Seq and Mass Spec where performed, 2 biological repeats are considered enough in a time-course as deviations from the time-course would be more noticeable (see below for estimate of variation and use of Pearson's correlation)
1.b. For animal studies, include a statement about sample size estimate even if no statistical methods were used.	N/A
2. Describe inclusion/exclusion criteria if samples or animals were excluded from the analysis. Were the criteria pre-established?	N/A
3. Were any steps taken to minimize the effects of subjective bias when allocating animals/samples to treatment (e.g. randomization procedure)? If yes, please describe.	N/A
For animal studies, include a statement about randomization even if no randomization was used.	N/A
4.a. Were any steps taken to minimize the effects of subjective bias during group allocation or/and when assessing results (e.g. blinding of the investigator)? If yes please describe.	The initial steps of the proteomics analysis was conducted by researchers who were blinded by the sample identities. The conditions were provided as numerical tube numbers, and the experimenters were not given information on the sample treatment/conditions
4.b. For animal studies, include a statement about blinding even if no blinding was done	N/A
5. For every figure, are statistical tests justified as appropriate?	Figure 2: Differential mRNA expression was defined as fold change > 1.5 and FDR < 0.2 (t test, Benjamini-Hochberg corrected) based on two independent experiments. Differential protein expression was defined as fold change > 1.15 and FDR < 0.2 (t test, Benjamini-Hochberg corrected) based on two independent experiments.
Do the data meet the assumptions of the tests (e.g., normal distribution)? Describe any methods used to assess it.	N/A

Is there an estimate of variation within each group of data?	We perform Pearson's correlation test between biological replicates. A pearson's correlation over 0.5 was the threshold to consider RNA or Protein dynamics of a given gene in the analysis
Is the variance similar between the groups that are being statistically compared?	N/A

C- Reagents

6. To show that antibodies were profiled for use in the system under study (assay and species), provide a citation, catalog number and/or clone number, supplementary information or reference to an antibody validation profile. e.g., Antibodypedia (see link list at top right), 1DegreeBio (see link list at top right).	Primary antibodies used (all 1:1000 unless otherwise stated): β-ACT (Sigma-Aldrich #A5316, 1:10,000 dilutions), BAX (CST #5023), DDB2 (CST #5416), KDM4A (CST #5328), MDM2 (Calbiochem #op46), p21 (CST #2947), p53 (Santa Cruz Biotechnology #sc-126, 1:5,000 dilutions), PPDM1D (Santa Cruz Biotechnology #sc-20712). Secondary antibodies used: anti-mouse IgG HRP-linked (Invitrogen #62-6520, 1:10,000 dilutions), anti-rabbit IgG HRP-linked (CST #7074, 1:10,000 dilutions)
7. Identify the source of cell lines and report if they were recently authenticated (e.g., by STR profiling) and tested for mycoplasma contamination.	MCF7 cells were gifted from Uri Alon Lab and have subsequently tested negative for mycoplasma and other microbial contamination. STR (short tandem repeat) profiling were also performed by Dana Farber Cancer Institute (DFCI) Molecular Diagnostics Laboratory to verify identity.

* for all hyperlinks, please see the table at the top right of the document

D- Animal Models

8. Report species, strain, gender, age of animals and genetic modification status where applicable. Please detail housing and husbandry conditions and the source of animals.	N/A
9. For experiments involving live vertebrates, include a statement of compliance with ethical regulations and identify the committee(s) approving the experiments.	N/A
10. We recommend consulting the ARRIVE guidelines (see link list at top right) (PLoS Biol. 8(6), e1000412, 2010) to ensure that other relevant aspects of animal studies are adequately reported. See author guidelines, under 'Reporting Guidelines'. See also: NIH (see link list at top right) and MRC (see link list at top right) recommendations. Please confirm compliance.	N/A

E- Human Subjects

11. Identify the committee(s) approving the study protocol.	N/A
12. Include a statement confirming that informed consent was obtained from all subjects and that the experiments conformed to the principles set out in the WMA Declaration of Helsinki and the Department of Health and Human Services Belmont Report.	N/A
13. For publication of patient photos, include a statement confirming that consent to publish was obtained.	N/A
14. Report any restrictions on the availability (and/or on the use) of human data or samples.	N/A
15. Report the clinical trial registration number (at ClinicalTrials.gov or equivalent), where applicable.	N/A
16. For phase II and III randomized controlled trials, please refer to the CONSORT flow diagram (see link list at top right) and submit the CONSORT checklist (see link list at top right) with your submission. See author guidelines, under 'Reporting Guidelines'. Please confirm you have submitted this list.	N/A
17. For tumor marker prognostic studies, we recommend that you follow the REMARK reporting guidelines (see link list at top right). See author guidelines, under 'Reporting Guidelines'. Please confirm you have followed these guidelines.	N/A

F- Data Accessibility

18: Provide a "Data Availability" section at the end of the Materials & Methods, listing the accession codes for data generated in this study and deposited in a public database (e.g. RNA-Seq data: Gene Expression Omnibus GSE39462, Proteomics data: PRIDE PXD000208 etc.) Please refer to our author guidelines for 'Data Deposition'.<br><br>Data deposition in a public repository is mandatory for:<br>a. Protein, DNA and RNA sequences<br>b. Macromolecular structures<br>c. Crystallographic data for small molecules<br>d. Functional genomics data<br>e. Proteomics and molecular interactions	Proteomics raw data and search results were deposited in the PRIDE archive (Perez-Riverol et al, 2019) and can be accessed under ProteomeXchange accession numbers: PXD027030 and are publicly available at http://www.ebi.ac.uk/pride/archive/projects/PXD027030.
19. Deposition is strongly recommended for any datasets that are central and integral to the study; please consider the journal's data policy. If no structured public repository exists for a given data type, we encourage the provision of datasets in the manuscript as a Supplementary Document (see author guidelines under 'Expanded View' or in unstructured repositories such as Dryad (see link list at top right) or Figshare (see link list at top right).	N/A
20. Access to human clinical and genomic datasets should be provided with as few restrictions as possible while respecting ethical obligations to the patients and relevant medical and legal issues. If practically possible and compatible with the individual consent agreement used in the study, such data should be deposited in one of the major public access-controlled repositories such as dbGAP (see link list at top right) or EGA (see link list at top right).	N/A
21. Computational models that are central and integral to a study should be shared without restrictions and provided in a machine-readable form. The relevant accession numbers or links should be provided. When possible, standardized format (SBML, CellML) should be used instead of scripts (e.g. MATLAB). Authors are strongly encouraged to follow the MIRIAM guidelines (see link list at top right) and deposit their model in a public database such as Biomodels (see link list at top right) or JWS Online (see link list at top right). If computer source code is provided with the paper, it should be deposited in a public repository or included in supplementary information.	Source code for the mathematical model is available at https://github.com/albajimenezasins/Proteomics_MSB_2022

G- Dual use research of concern

22. Could your study fall under dual use research restrictions? Please check biosecurity documents (see link list at top right) and list of select agents and toxins (APHIS/CDC) (see link list at top right). According to our biosecurity guidelines, provide a statement only if it could.	N/A
